# Using physical property surrogate models to perform accelerated multi-fidelity optimization of force field parameters†

**DOI:** 10.1039/d2dd00138a

**Published:** 2023-05-05

**Authors:** Owen C. Madin, Michael R. Shirts

**Affiliations:** a Department of Chemical & Biological Engineering, University of Colorado Boulder Boulder CO USA 80309 michael.shirts@colorado.edu

## Abstract

Accurate representations of van der Waals dispersion–repulsion interactions play an important role in high-quality molecular dynamics simulations. Training the force field parameters used in the Lennard Jones (LJ) potential typically used to represent these interactions is challenging, generally requiring adjustment based on simulations of macroscopic physical properties. The large computational expense of these simulations, especially when many parameters must be trained simultaneously, limits the size of training data set and number of optimization steps that can be taken, often requiring modelers to perform optimizations within a local parameter region. To allow for more global LJ parameter optimization against large training sets, we introduce a multi-fidelity optimization technique which uses Gaussian process surrogate modeling to build inexpensive models of physical properties as a function of LJ parameters. This approach allows for fast evaluation of approximate objective functions, greatly accelerating searches over parameter space and enabling the use of optimization algorithms capable of searching more globally. In this study, we use an iterative framework which performs global optimization with differential evolution at the surrogate level, followed by validation at the simulation level and surrogate refinement. Using this technique on two previously studied training sets, containing up to 195 physical property targets, we refit a subset of the LJ parameters for the OpenFF 1.0.0 (Parsley) force field. We demonstrate that this multi-fidelity technique can find improved parameter sets compared to a purely simulation-based optimization by searching more broadly and escaping local minima. Additionally, this technique often finds significantly different parameter minima that have comparably accurate performance. In most cases, these parameter sets are transferable to other similar molecules in a test set. Our multi-fidelity technique provides a platform for rapid, more global optimization of molecular models against physical properties, as well as a number of opportunities for further refinement of the technique.

## Abstract 2

We present a multi-fidelity method for optimizing nonbonded force field parameters against physical property data. Leveraging fast surrogate models, we accelerate the parameter search and find novel solutions that improve force field performance.

## Graphical abstract

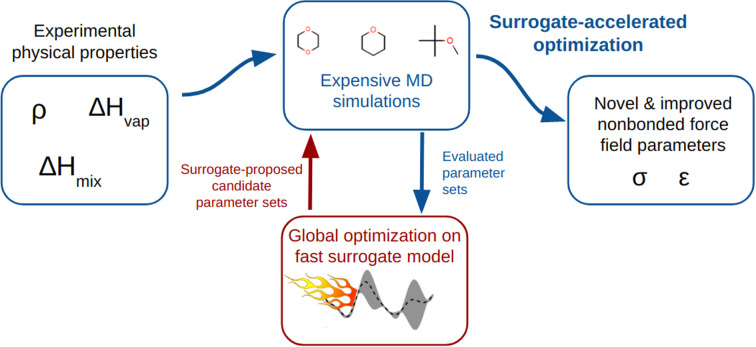

## 1 Introduction

### 1.1 Accurate force fields are important in computational biophysics

Accurate molecular interaction potentials, usually referred to as force fields, are an essential part of modern molecular dynamics workflows. For common applications such as simulations of proteins and computer aided drug design (CADD), the simple fixed-charge force field functional form^1–3^ is generally used. This formulation splits the potential energy of molecules into discrete components, with separate energy terms for each component.^4^ Broadly, these can be divided into the bonded (or valence) components, which give the energies corresponding to the bond lengths, bond angles, and torsional angles, and the non-bonded components, representing short-range dispersion–repulsion interactions and longer-range coulombic interactions.

This type of force field has been successful in many applications because of its simplicity, interpretability, and computational efficiency. Many studies have used these force fields to probe the mechanisms of protein dynamics,^5–8^ and they have become widely adopted in the pharmaceutical industry as a means of screening drug candidate molecules *in silico*.^8–12^ While these force fields are quite simple in their functional form, their accuracy is dependent on hundreds to thousands of empirical parameters, which dictate the strength of interactions in different molecular configurations and in different chemical environments. Decades of effort from the computational chemistry community have produced many different parameter sets to cover a wide range of chemistries,^3,13–16^ largely by fitting parameters to quantum mechanics (QM) calculations^17,18^ and experimental physical properties.^19,20^

### 1.2 Non-bonded training is expensive and difficult

Over the years, fitting of the bonded parameters has perhaps received the most attention, due to their importance in determining the internal structure of molecules and proteins, and the relative ease of generating gas-phase QM data for the molecules of interest. Fitting the atomic partial charges used in the coulombic potential has also received significant attention, but is slightly more difficult, as mapping an continuous electrostatic potential onto a set of discrete atoms is conformation-dependent and involves a loss of fidelity. However, modelers have achieved good results using QM-based methods such as RESP^21,22^ and semi-empirical methods such as AM1-BCC.^23,24^

The dispersion–repulsion interactions, usually modeled with the Lennard-Jones (LJ) potential, have received the least attention in fitting, as they are typically trained against experimental physical property data,^19,20,25^ since obtaining dispersion–repulsion estimates from QM is difficult.^26^ This leads to challenges with curating appropriate sets of experimental physical property data from the literature, as well as the computational cost of simulating sets of physical property data with molecular dynamics. Most physical properties used in training, which include densities,^19^ enthalpies of vaporization,^19^ enthalpies of mixing,^27^ solvation free energies^20^ and dielectric constants,^28,29^ require equilibrium simulations in one or more phases, and in some cases may require alchemical simulation techniques.^30^ In conjunction with the need to train against larger datasets to ensure accuracy and transferability, this makes optimization of LJ parameters a challenging problem. Calculating a single objective function value in order to measure parameter fitness requires a large number of simulations, which can be difficult to coordinate and execute, especially depending on available computational resources. As a result, one can find many instances of LJ parameters in major force fields that have remained unchanged for more than 20 years, despite significant advancements in hardware, simulation software, and methodology in that time.

Recently, as part of the Open Force Field (OpenFF) Initiative, we have examined new methods of LJ parameter optimization. Central to these efforts is the development of the OpenFF Evaluator simulation workflow driver,^31^ which provides a standardized set of workflows for automatically building and executing physical property simulations for a given training or test data set. With the automation that this software provides, we can apply optimization techniques to LJ parameters with minimal human intervention. In particular, this software enabled the application of the ForceBalance^32^ parameter optimization package to improve LJ parameters. Using regularized least squares optimization with the L-BFGS-B algorithm,^33^ we minimized an objective function that captures the ability of a parameter set to reproduce physical property observables. Using this framework, we also studied the benefits of including physical property data of mixtures in training LJ parameters,^27^ then applied that training method to a production force field, OpenFF 2.0.0 (also known as “Sage”).^34^

While this approach has produced parameter sets with improved performance in predicting experimental physical properties, using simulation-based regularized least-squares optimization has significant limitations. A major drawback is that, regardless of the optimization algorithm used, the number of objective function evaluations possible is limited by the computational cost of simulations. This limits the number of parameter sets that can be considered during optimization, making it difficult to explore high-dimensional and complex parameter spaces. This also necessitates the use of cheaper, fully local optimization methods such as L-BFGS-B with termination after a set number of steps.^27^ When coupled with a regularization term in the objective function, included both to ensure the stability of the optimization and to guard against overfitting,^32^ local optimization methods have a high probability of remaining in any local minima dictated by its initial values. This means that our ability to explore new areas of parameter space that may provide significant improvement is blunted because of the expense of evaluating the objective and the difficulty of escaping a local minima with a gradient-based optimization method.

### 1.3 Surrogate modeling can accelerate non-bonded training

To facilitate faster evaluation of complex objective functions, modelers often use surrogate models,^35,36^ which are meant to approximate an expensive function with a simpler alternative that captures a sufficient amount of the important information of the response function. Surrogate modeling techniques have been developed in response to diverse sets of scientific and engineering challenges, including geological modeling,^37,38^ engineering design,^39,40^ and chemical process modeling.^41,42^ A popular technique is Gaussian process (GP) surrogate modeling, which has seen adoption in many disciplines^43–45^ due to its simplicity and efficacy in data-sparse regimes. While these surrogates cannot be perfect imitations of the high-level responses, for sufficiently smooth functions, we can construct surrogates with a reasonable level of accuracy with only a limited number of expensive evaluations. In the context of molecular simulation parameter optimization, Befort *et al.*^46^ demonstrated a method of optimizing LJ parameters by building GP surrogates based on physical properties and applied this method to several hydrofluorocarbons as well as ammonium perchlorate.

In this paper, we build on this approach, as well as engineering optimization literature^47^ and our OpenFF Evaluator software, to introduce a multi-fidelity optimization framework based on the construction of Gaussian process (GP) surrogate models that approximate the response surface of many physical properties with respect to changes in the LJ parameters. Using the accelerated objective evaluation offered by the surrogates, we implement a global optimization algorithm to search broadly and propose candidate parameter sets. We then validate these parameter sets by evaluating the objective at the simulation level, accepting candidates in good agreement. Iterating between global optimization over the surrogate, and simulation-level validation and surrogate refinement, we can drive the optimizer to explore promising regions of parameter space with a limited number of simulation evaluations.

We test this approach by performing multi-fidelity LJ optimization for 12 commonly exercised LJ parameters from the OpenFF 1.0.0 (Parsley) force field. In training, we use a set of 56 pure compound physical properties curated in a previous paper.^27^ We also benchmark the results against test sets curated in the same paper; while newer versions of OpenFF exist, using OpenFF 1.0.0 allows a direct comparison to the results of our previous optimization. With this context, we characterize the optimization method, and discuss reproducibility, seed configurations, and optimization trajectories. We also show that this method can be extended to larger problems by applying it to a larger training set of 195 mixture properties from the same paper.

## 2 Methods

### 2.1 Optimization strategy

Our optimization strategy aims to minimize an objective function *χ*(*θ*) as in eqn (1), where *θ* is a vector of force field parameters, and *χ* is some measure of the fitness of those parameters.1
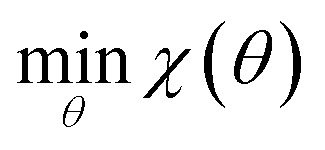
In our applications, parameter fitness is described by the ability of a force field containing those parameters to reproduce a specific training set of experimental physical properties, although we note that this strategy could also be applied to a training set containing quantities from QM simulations. The optimization strategy we employ is adapted from the framework proposed by Dennis and Troczon^47^ and features two levels of fidelity for estimating the objective function for a parameter set:

• “Simulation level”, where the objective function is directly evaluated by using molecular dynamics to simulate the training set with a force field containing the parameter set. This is considered to be the “ground truth”, as it is a direct measurement the force field's performance, although there is some level of statistical uncertainty due to the stochasticity in the molecular dynamics simulation.

• “Surrogate level”, where the objective function is estimated by a collection of surrogate models that approximate the result of a simulation-level evaluation of the training set. The surrogate-level evaluation of the objective function has systematic uncertainty where the surrogates approximation deviates from the simulation-level estimation of the training set. It may also have statistical uncertainty depending on the type of surrogate used, although the surrogates that we use do not.

**Fig. 1:**
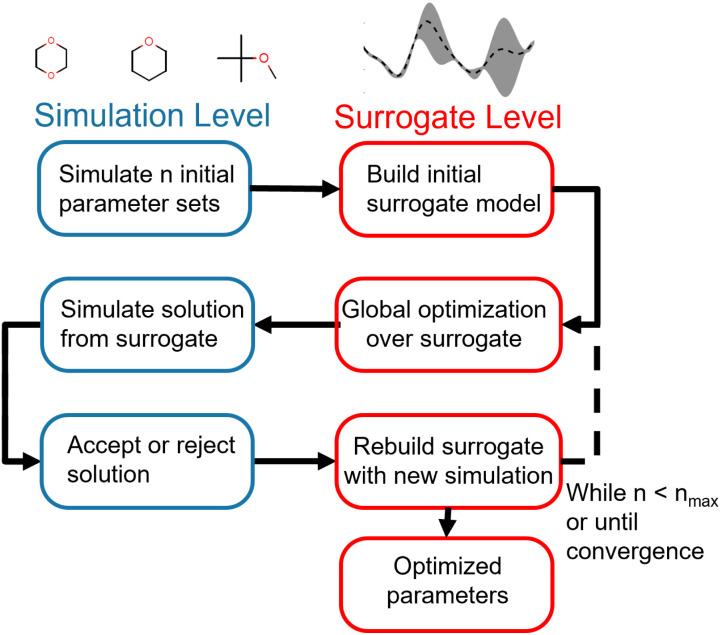
Flowchart of multi-fidelity optimization strategy. Optimization is initialized by simulating an initial sample of parameter vectors. Surrogate models for each of the physical properties in the training set are then built from this initial sample. Global optimization is then performed at the surrogate level, utilizing the speedup gained with surrogate-level fast objective evaluation. Once this proposes a candidate vector of optimized parameters, the objective function for that parameter vector is evaluated at the simulation level. If the simulation-level objective is lower than the simulation objective for the previous parameter vector, the new parameter vector is accepted as an improved solution; if not, it is rejected. Regardless of acceptance or rejection, the surrogate model is rebuilt with the information from the simulation-level evaluation. This process is then repeated until a maximum number of simulation optimizations is reached, a convergence criteria is met, or the optimizer cannot find an improved solution.

Our optimization strategy relies on the cheaper but less accurate surrogate level to perform most of the optimization, using the more accurate and expensive simulation level only to build the surrogates and validates proposed surrogate-level solutions. The optimization framework is illustrated in Fig. 1.

The advantage of this strategy is its use of the properties of both the surrogate and simulation level to drive optimization. While surrogate level evaluation is much faster than simulation-level evaluation, surrogates need simulation points in the region of interest to accurately reproduce the objective function. Since parameter spaces are large and the region of interest is not known *a priori*, an exhaustive strategy would require a very large number of simulation-level evaluations to build globally accurate surrogates, negating the speedup gained by using surrogates. We instead build a surrogate from a minimal initial set of evaluations of the objective function and allow the surrogate to suggest new parameter vectors for the simulation level to evaluate. We can therefore iteratively drive the optimization towards the region of interest without incurring too much computational cost, acquiring more information to improve the surrogates along the way. This allows us to pair the global optimization strategies available at the surrogate level with the accuracy of simulation-level validation. While this overall strategy is not strictly a global optimization, since we only use global optimizations at the approximate surrogate level, it does allow for a much wider search of the parameter space than a gradient-based local optimization.

This strategy is sufficiently general to allow for the use of a large variety of objective functions, surrogate-level global optimization techniques, and surrogate modeling strategies. In this particular study we focus on a single combination: a weighted least-squares objective function based on experimental properties, the differential evolution global optimization algorithm, and Gaussian process (GP) surrogate modeling.

#### 2.1.1 Objective function

The objective function we use, shown in eqn (2), is adapted from the type used in the ForceBalance optimization software package and used in our previous work.^27^ While that objective included a regularization term for stability and to prevent overfitting, we omit that term, allowing our algorithm to search more broadly to find optimized parameter vectors.2
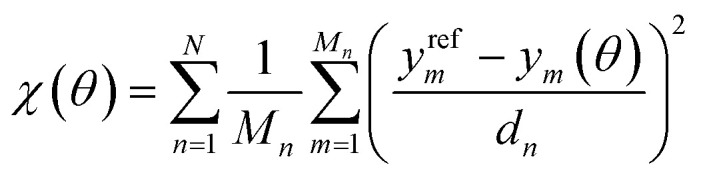


In this equation, we consider *N* types of physical properties, each with some number *M*_*n*_ of measurements for that type. The quantity *y*_*m*_ represents the value of the *m*th measurement for a physical property type, and the denominator *d*_*n*_ is a scaling coefficient for a given property. The values of *y*_*m*_(*θ*) can either be obtained directly from simulation, or from surrogate models based on those simulations. The scaling coefficients are set so that each physical property type contributes equally to the objective function for OpenFF 1.0.0,^18^ the starting point of our optimizations.

#### 2.1.2 Global optimization

We use the differential evolution^48^ global optimization algorithm, as implemented in the SciPy Python package version 1.7.0.^49^ Differential evolution is a stochastic direct search algorithm similar to other genetic algorithm strategies.^50^ In this strategy, a set of *N* initial vectors is proposed, and then “mutated” by randomly increasing or decreasing elements of the vector, and recombined, by randomly replacing some elements of the vector with elements of other vectors. The objective function is then evaluated for each of the proposed vectors, and a new set of vectors is proposed based on the lowest objective functions. This process is repeated until convergence, when new vectors no longer outperform the current solutions. We use the default optimization parameters in SciPy, with a population size of 180 vectors, an iteration-dependent mutation constant selected from the range (0.5, 1), and a recombination constant of 0.7. We note that, depending on the problem, a single iteration of this algorithm requires between 10^3^ and 10^4^ objective function evaluations *per iteration*, each of which depends on the value of 50–200 physical properties.

The bounds of the global optimization over the surrogate model are determined by the parameter sets used to build the surrogate. For each parameter *θ*_*i*_ in the parameter vector *θ*, the bounds for *θ*_*i*_ are determined by the minimum (min(*θ*_*i*_)) and maximum value (max(*θ*_*i*_)) of *θ*_*i*_ in the set of parameter vectors *Θ* = [*θ*^1^, *θ*^2^, …, *θ*^*N*^] used to build the surrogate. We then apply a small expansion factor *η* to the parameter range, so that the optimization algorithm can search outside of the initial simulation box (described in Section 2.4.2). To expand the box, we multiply max(*θ*_*i*_) by *η*, and divide min(*θ*_*i*_) by *η* to form the bounds box [LB(*θ*_*i*_), UB(*θ*_*i*_)]. We chose the value of *η* to be 1.1, expanding the box 10% in each direction, to allow the optimizer to search aggressively. This process is repeated for each parameter *θ*_*i*_ ∈ *θ* to form an *N*-dimensional box, and is described in eqn (3). Due to the nature of the optimization (as described in Fig. 1), the bounds are recomputed at each iteration, after more simulation information has been added to the surrogate. This allows the bounds to change significantly over the optimization as the algorithm explores new areas of parameter space.3
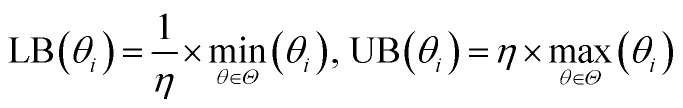


### 2.2 Construction of physical property surrogates

GP surrogate models are built with the BoTorch^51^ software package, version 0.6.0, which provides a convenient and extensible framework for building a large number of surrogates. Surrogates are constructed individually for each physical property in the test set, from all of the simulation level evaluations available; *e.g.* if there are simulations of 20 physical properties with 10 different LJ parameter vectors, then our process builds 20 individual surrogate models, each using all 10 parameter vectors in their construction. Objective functions are calculated from the surrogates' predictions of their respective physical properties; the surrogate does not predict the objective function directly, such as is done in Bayesian optimization. All surrogates use a constant mean function and RBF (radial basis function) covariance kernel; independent length scales *l* for each parameter are chosen using automatic relevance determination (ARD).^52^ The length scales *l* in the covariance kernel represent the distances over which points are correlated in each dimension.

If a simulation of a physical property at a given set of parameters finishes with errors, that set is omitted from surrogate building; additionally, any sets that have density measurements lower than 20% of the experimental value are omitted, as this likely indicates that the parameters have induced a phase change. The rationale for this criteria is that we are attempting to build a surrogate model which accurately predicts liquid densities in over parameter space, and parameter sets that predict a gaseous or solid system at temperature and pressure where that system should be liquid will not provide useful information. However, this restriction did not affect the optimization, as parameters violating the density constraint were never produced through our optimizations.

In cases where an optimization iteration over a surrogate model fails to find a lower objective value than the current simulation objective, surrogates are rebuilt with constraints on the length scales *l* used for the variances of each parameter. This approach was chosen as we found in testing that optimizations may fail because a surrogate was set with a length scale too low during ARD, producing a surrogate with poor quality for a particular physical property. If the optimizer cannot find a better objective value over the surrogate, it is first rebuilt with a length scale constraint such that *l* > 10^−10^; if this is not successful, a stricter length scale constraint of *l* > 10^−5^ is imposed. If this is still not successful, the optimization is terminated. The quality of the surrogate model is reduced when length scale constraints are introduced, so constraints are not used unless an optimization fails.

### 2.3 Physical property simulations

Physical property simulations were handled with the OpenFF Evaluator^31^ software package, version 0.3.4,^53^ using the default workflows^54^ for all properties simulated. We performed simulations to estimate pure density (*ρ*_L_), mixture density (*ρ*_L_(*x*)), enthalpy of vaporization (Δ*H*_vap_) and enthalpy of mixing (Δ*H*_mix_(*x*)). To summarize the procedure, we performed all condensed-phase simulations in the NPT ensemble, with initial simulation boxes of 1000 molecules built using PackMOL.^55^ After building the boxes, we perform an energy minimization on the simulation boxes, followed by a 0.2 ns equilibration simulation and a 2 ns production simulation, which was found to be sufficient to converge these simple physical properties.^31^ In the calculation of Δ*H*_vap_, we use 30 ns single molecular NVT simulations without periodic boundary conditions to estimate the gas phase energies. All simulations use a 2 fs timestep and a Langevin integrator with BAOAB splitting.^56^ More complete simulation details are available in our previous work,^27^ which uses the same simulation workflows.

### 2.4 Optimization tasks

We focused on two separate optimization tasks, both developed in our previous study.^27^ Both tasks optimize the same set of 12 LJ parameters (*R*_min/2_ and *ε* for 6 LJ SMIRKS types), and both use the same small molecules (alkanes, alcohols, ethers, esters and ketones) in the their training sets. The tasks are differentiated by the different types of physical property training data that are used in the evaluation of the objective function:

(1) “Pure only”: this task optimizes against a set of 56 pure compound measurements, *ρ*_L_ and Δ*H*_vap_ for each of 28 compounds in the training set. We used this task to test the optimization strategy, as it represents the typical type of training set used in LJ optimization, and has relatively low computational expense because of the number and types of physical properties that need to be estimated.

(2) “Mixture only”: this task optimizes against a larger set of 195 physical properties of binary mixtures (Δ*H*_mix_(*x*) and *ρ*_L_(*x*)). This task extends the strategy to a significantly larger training set, and represents the type of training set that performed best in our previous study.

While we reported optimized parameter sets for these tasks in our previous work, here we use those sets as a baseline to test our multi-fidelity strategy.

#### 2.4.1 Parameters to be optimized

We optimize the LJ *R*_min/2_ and *ε* for 6 LJ types, which are described in Table 1. We also note that several LJ types are exercised by molecules in the training set, but are not optimized, due to either having very specific chemical contexts that are not exercised widely enough to optimize, or, in the case of the [#1:1]-[#8] (hydroxyl hydrogen) parameter, because the *ε* has been set to an arbitrary small non-zero value to avoid unphysical effects.^57^

**Table 1:** All LJ SMIRKS types, both adjusted and not adjusted, in the training of OpenFF 2.0.0, along with descriptions of the chemical contexts they describe. Both LJ *ε* and *R*_min/2_ are adjusted for each of the types under the “refitted parameters” subheading

Refitted SMIRKS type	Description
**Refitted parameters**	
[#1:1]-[#6X4]	Hydrogen attached to tetravalent carbon
[#6:1]	Generic carbon
[#6X4:1]	Tetravalent carbon
[#8:1]	Generic oxygen
[#8X2H0+0:1]	Divalent oxygen with no hydrogens attached
[#8X2H1+0:1]	Divalent oxygen with one hydrogen attached

**Parameters exercised but not refitted**	
[#1:1]-([#6X4])	Hydrogen attached to tetravalent carbon attached to an electronegative atom
-[#7, #8, #9, #16, #17, #35]	
[#1:1]-[#6X3]	Hydrogen attached to trivalent carbon attached to 2 electronegative atoms
(∼[#7, #8, #9, #16, #17, #35])	
∼[#7, #8, #9, #16, #17, #35]	
[#1:1]-[#8]	Hydrogen attached to oxygen

#### 2.4.2 Initial physical property simulations

To build an initial surrogate in each optimization, we simulate an initial set of *N* parameter vectors, one of which is always the parameter vector corresponding to OpenFF 1.0.0. We select these vectors from an initial parameter space, described in Table 2. This space is measured in percentage of the parameter values from OpenFF 1.0.0, and is determined from the results of our previous optimization study, based on how much each parameter was adjusted in that study. From this space, we select the remaining *N* − 1 parameter vectors using Latin hypercube sampling (LHS), as implemented with the Surrogate Modeling Toolbox^58^ (SMT) Python library, version 1.1.0. Since the optimization bounds are recomputed after each optimization iteration, solutions are not restricted to this initial space.

**Table 2:** Parameter space that initial parameter vectors are sampled from, defined as percentages of OpenFF 1.0.0 values. For a set of *N* parameter vectors used to initialize the surrogate model, Latin hypercube sampling is used to select *N* − 1 parameter vectors from this parameter space, with the final parameter vector being the parameters from OpenFF 1.0.0

Refit parameters		
Refit SMIRKS type	*ε* initial parameter range (% of OpenFF 1.0.0)	*R*_min/2_ initial parameter range (% of OpenFF 1.0.0)
[#1:1]-[#6X4]	(50, 150)	(95, 105)
[#6:1]	(90, 110)	(95, 105)
[#6X4:1]	(90, 110)	(95, 105)
[#8:1]	(95, 105)	(95, 105)
[#8X2H0+0:1]	(95, 105)	(95, 105)
[#8X2H1+0:1]	(95, 105)	(95, 105)

#### 2.4.3 “Pure only” optimization task

**Fig. 2:**
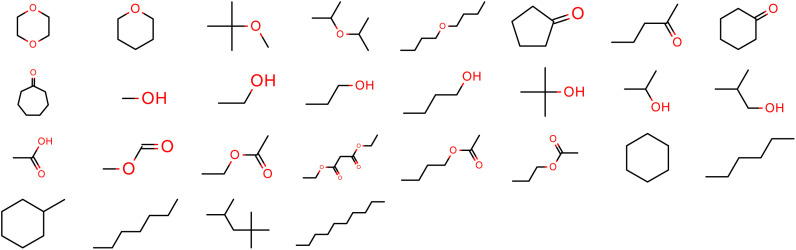
Molecules in the “pure only” training set. Physical properties in this set include one measurement each of *ρ*_L_ and Δ*H*_vap_, and are sourced from either the NIST ThermoML Archive (*ρ*_L_) or hand-curated from literature (Δ*H*_vap_).

The “pure only” optimization task fits the LJ parameters against a total of 56 physical properties (*ρ*_L_ and Δ*H*_vap_ for a set of 28 molecules), which are shown in Fig. 2. A list of the molecules in the “pure only” training set are available in the ESI, Section S1.1.†

The measurements here are either sourced from the NIST ThermoML Archive^59,60^ (*ρ*_L_) or hand-curated from literature (Δ*H*_vap_),^61–72^ because of the low number of Δ*H*_vap_ data points in the ThermoML Archive. All measurements are selected at temperatures and pressures close to ambient (∼1 atm, 273.15–318.15 K).

For this optimization task, we performed optimizations using *N* = 5 and *N* = 10 initial points, to test the effect of the number of initial simulation points on the performance of the algorithm. We performed 5 replicates for both *N* = 5 and *N* = 10 initial points, in order to assess the consistency of the algorithm. For the *N* = 10 replicates, a different set of 9 LHS initial points is selected each time; for the *N* = 5 replicates, each set of initial points is formed by subsampling 4 LHS points from one of the *N* = 10 replicate initial sets, in order to minimize simulation expense.

#### 2.4.4 “Mixture only” training set

**Fig. 3:**
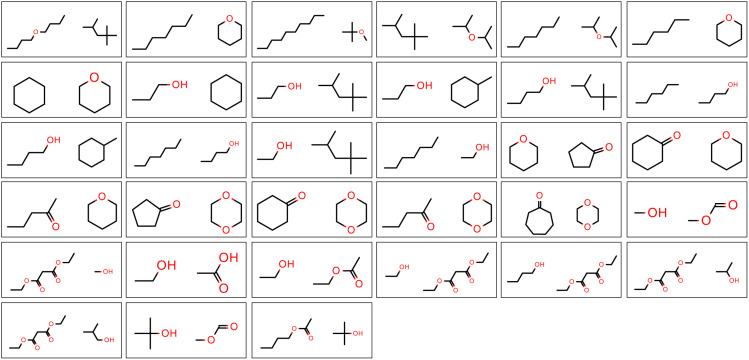
Molecules in the “mixture only” training set. Physical properties in this set include measurements of *ρ*_L_(*x*) and Δ*H*_mix_(*x*) at conditions close to ambient (∼1 atm, 273.15–318.15 K), and several concentrations ((*x*_1_ = 0.25, *x*_2_ = 0.75), (*x*_1_ = 0.5, *x*_2_ = 0.5), (*x*_1_ = 0.75, *x*_2_ = 0.25)), where available, yielding a total of 195 measurements. All measurements are sourced from the NIST ThermoML Archive.

The “mixture only” optimization task optimizes the LJ parameters against a set of 195 physical properties (*ρ*_L_(*x*), Δ*H*_mix_(*x*)) for the set of molecule pairs shown in Fig. 3. These molecule pairs are drawn from the same set of molecules as used in the “pure only” training set. All measurements here are selected from the NIST ThermoML archive, and are selected at temperatures and pressures close to ambient (∼1 atm, 273.15–318.15 K). We select measurements at concentrations within 0.05 mole fraction of 3 target concentrations for each mixture, where available: (*x*_1_ = 0.25, *x*_2_ = 0.75), (*x*_1_ = 0.5, *x*_2_ = 0.5), (*x*_1_ = 0.75, *x*_2_ = 0.25). If no measurements are available within 0.05 mole fraction of a target concentration, no data point is selected for that target concentration. A list of the mixtures in the “mixture only” training set is available in the ESI, Section S1.2.†

For this second optimization task, we performed an optimization using *N* = 20 initial points, due to the increased complexity of the training set. After this optimization, we performed a second optimization using *N* = 10 initial points, in order to test whether a more data-sparse optimization could be successful. For the *N* = 10 replicate, 9 initial points are subsampled from the 19 LHS points used in the *N* = 20 replicate to minimize simulation expense.

### 2.5 Benchmarking

To assess the quality and transferability of the parameter sets produced by our optimization, we tested them on a set of physical properties (29 *ρ*_L_, 318 *ρ*_L_(*x*), 29 Δ*H*_vap_, and 236 Δ*H*_mix_(*x*)) for a new set of molecules and molecule pairs, which serves as the test set. This data set was curated for our previous work, and its selection and composition are discussed there.^27^ Physical properties in this set are either hand-curated from literature (*ρ*_L_, Δ*H*_vap_), or are selected automatically from the NIST ThermoML Archive. Benchmarking simulations are performed using the same OpenFF Evaluator workflows as simulations used in the optimization process.

## 3 Results & discussion

### 3.1 Pure training set

#### 3.1.1 Optimization

Optimization was generally successful with both *N* = 5 and *N* = 10 initial parameter vectors, as the process reached significantly lower objective function values than the initial force field in every case. Additionally, when comparing training set RMSE for Δ*H*_vap_, all optimization replicates significantly outperform the regularized least squares optimization.

Out of the 10 optimizations run, 4 of them terminated early after the surrogate optimizer could not find an improved solution. This is related to the issues with ARD noted in Section 2.2. This suggests that further refinement is needed to improve the robustness of the surrogate model. Optimizations used between 15–25 total simulations, compared to the 12 used in the simulation-only optimization.

**Fig. 4:**
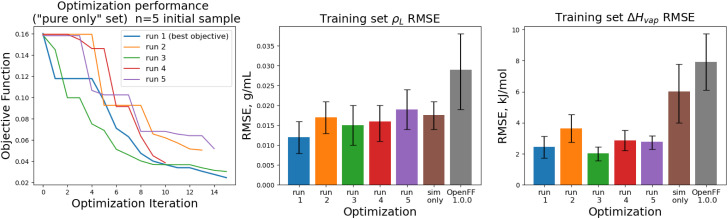
Performance of the multi-fidelity optimization algorithm on the “pure only” training set, for replicates run with *N* = 5 initial parameter vectors. Left panel shows the objective function at each iteration of the optimization. Right two panels show the training set RMSE for *ρ*_L_(*x*) and Δ*H*_mix_(*x*) for each of the optimizations, as well as OpenFF 1.0.0 and the previous simulation-only optimization (labeled “sim only” in the graphs). Error bars represent 95% confidence intervals, computed with bootstrapping over the set of molecules in the training set.

The objective function trajectories and training set RMSEs of the replicates starting from *N* = 5 initial points are shown in Fig. 4, with the training set RMSEs of OpenFF 1.0.0 and the optimized set from our previous work shown for comparison.

We see that in most cases, the optimizer struggles initially, with a high percentage of proposed solutions rejected in the first 8 steps. While these steps do not immediately yield an improved force field, the parameter vectors they propose are added to the pool of parameter vectors used to build surrogates, eventually exploring enough space to find an improved solution, with an average objective of 0.039 *vs.* an initial objective of 0.16, an average *ρ*_L_ training set RMSE of 0.016 g mL^−1^ (initial RMSE 0.027 g mL^−1^), and an average Δ*H*_vap_ RMSE of 2.75 kJ mol^−1^ (initial RMSE 7.15 kJ mol^−1^).

**Fig. 5:**
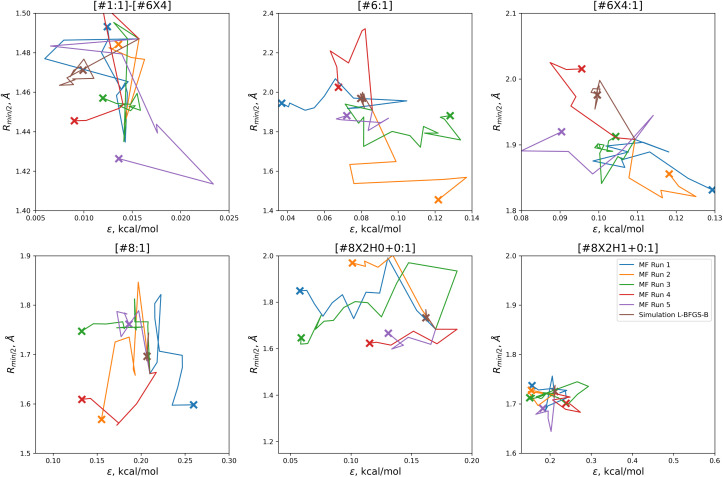
Parameter-space optimization trajectories for each of the replicates run with 5 initial parameter vectors, as well as the trajectory for the previous simulation-only optimization (brown). Trajectories show that our optimization technique searches widely and finds many distinct solutions for this optimization problem. Plot limits are shared with Fig. 7 for ease of comparison.

In order to find these improved solutions, the optimization algorithm searches widely and finds a number of qualitatively distinct minima. The optimization trajectories in parameter space, as well as the trajectory from the simulation-only optimization against the same training set, are shown in Fig. 5.

In comparison to the simulation-only optimization, shown in brown, the replicates of our multi-fidelity optimization search the parameter space much more broadly. Particularly, the values of oxygen and carbon parameters stay within a narrow range in the simulation-only optimization, but vary widely with our multi-fidelity technique.

**Fig. 6:**
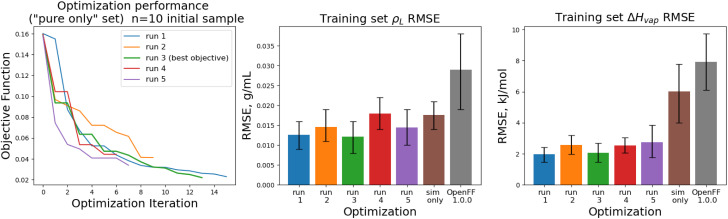
Performance of the optimization algorithm on the “pure only” training set, for replicates run with *N* = 10 initial parameter vectors. Left panel shows the objective function at each iteration of the optimization. Right two panels show the RMSE for Δ*H*_mix_(*x*) and *ρ*_L_(*x*) for the two optimizations, as well as OpenFF 1.0.0 and the previous simulation-only optimization (labeled “sim only” in the graphs). Error bars represent 95% confidence intervals, computed with bootstrapping over the set of molecules in the training set.

The training set RMSEs and objective functions for the *N* = 10 runs are shown in Fig. 6.

We note that the optimizations initialized with *N* = 10 initial parameter vectors improve the objective function with fewer iterations that the *N* = 5 optimizations. The *N* = 10 optimizations leverage the additional initial information to build more accurate surrogates, finding improved parameter sets sooner (at the expense of higher inital cost). The effectiveness of the optimization is also slightly improved over the *N* = 5 optimizations, with an average objective function of 0.031 (*N* = 5: 0.039), an average *ρ*_L_ training set RMSE of 0.014 g mL^−1^ (*N* = 5: 0.016), and an average Δ*H*_vap_ RMSE of 2.38 kJ mol^−1^ (*N* = 5: 2.75 kJ mol^−1^).

**Fig. 7:**
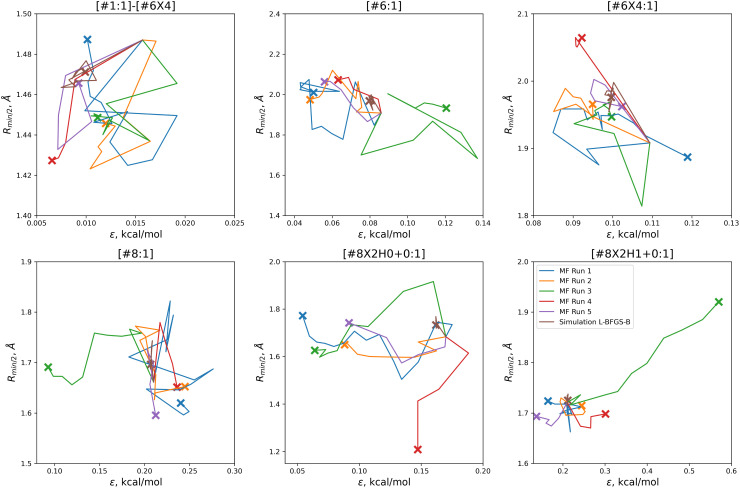
Optimization trajectories in parameter space for each of the replicates run with *N* = 10 initial parameter vectors. Trajectories show that our *N* = 10 initial parameter vector optimization technique searches more widely than our *N* = 5 technique, but is also more likely to drastically alter a parameter while deeply exploring a potentially promising candidate, as shown for runs 3 (green) and 4 (red). Plot limits are shared with figure for ease of comparison.

With the exception of two of the runs, the parameter trajectories in the initial *N* = 10 optimizations explore a similar range of parameters to the *N* = 5 optimizations, as shown in Fig. 7. In contrast, runs 3 and 4 make very large changes to some of the parameters, drastically deviating from the initial parameter set.

Particularly in the oxygen parameters for runs 3 (green) and 4 (red), we can see that these optimizations can make some very large parameter changes; run 3 has the lowest overall objective, but more than doubles the hydroxyl oxygen *ε*. This may suggest that adding some regularization could benefit the transferability of the optimization, but it also reflects that the ratio of targets to inputs (56:12) leads to an optimization where many solutions can be found.

#### 3.1.2 Parameter interpretation

**Fig. 8:**
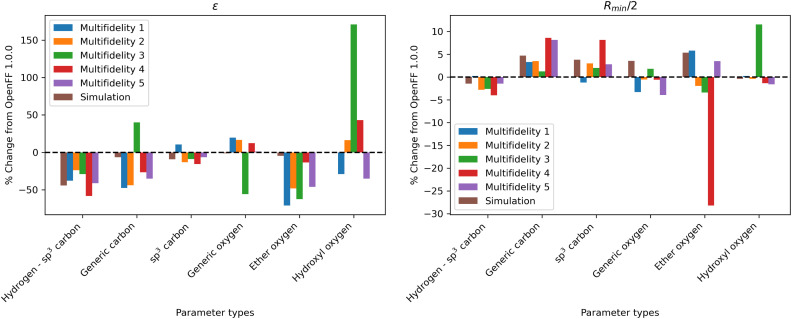
Changes in parameter values after optimization against the “pure only” data set, relative to OpenFF 1.0.0 (the initial values of the optimization), for the *N* = 10 optimization replicates, as well as the simulation-only solution previously obtained. Parameter changes are typically larger in multi-fidelity optimization compared to the simulation-only optimization, indicating improved exploration of the parameter space; however, this also leads to outliers (hydroxyl oxygen *ε*, ether oxygen *R*_min_/2).

**Fig. 9:**
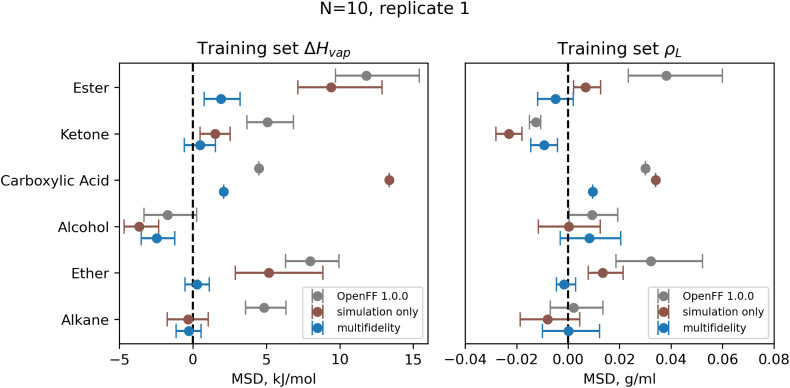
Bias in training set Δ*H*_vap_ and *ρ*_L_ by chemical functionality, as measured by the mean signed deviation (MSD), for OpenFF 1.0.0 and retrained parameters from *N* = 10 multi-fidelity run 1. Training with multi-fidelity optimization reduces or eliminates bias in several chemical functionalities, including alkanes, ketones, and ethers, where MSD is near 0 kJ mol^−1^. Reduction in MSD shown for run 1 is typical of all “pure only” *N* = 10 multi-fidelity runs; training set MSDs and RMSEs for all multi-fidelity optimization are available in ESI, Sections 2.1–2.3.† Error bars represent bootstrapped 95% confidence intervals.

Since the optimizations find diverse solutions, and the set of parameters is small enough to be reasonably interpretable, it is worth examining some of the parameter changes to understand their physical basis and inform future parameter fitting. Here we analyze some of the most notable changes from the *N* = 10 replicates, which had lower objective functions relative to the *N* = 5 replicates. The change in parameters from the original OpenFF 1.0.0 is shown in Fig. 8.To identify what points in the training dataset the parameter changes are affecting, we examine the bias of the physical properties training dataset, as measured by mean signed deviation (MSD) from experiment. To avoid confusion, we use the acronym MSD to refer to this bias, while RMSE refers to the root mean squared error. The bias before and after training for each chemical group is plotted for multi-fidelity run 1, shown in Fig. 9. Run 1 is shown as an example as it has one of the best objective functions and no unusual parameter changes (such as occurred in runs 3 and 4), and MSD values are similar for all optimization runs. Similar plots of RMSE and MSD for all 5 runs are available in the ESI, Section S2.2.†

One consistent trend in most optimizations is the significant overall reduction of *ε* for most parameter types. By decreasing the *ε*′s, cohesive forces in the liquid phase are reduced, lowering the barrier for “liberating” a molecule from the gas phase and thereby lowering the enthalpy of vaporization. In OpenFF 1.0.0, the enthalpy of vaporization measurements in the training set have a positive bias (MSD) of 5.03 kJ mol^−1^, with all moieties except alcohols having a positive deviation from experiment. After multi-fidelity optimization, the training sets have an average MSD (across all multi-fidelity runs) of 0.22 kJ mol^−1^.

The trend of reduced *ε*′s is strongest for the [#1:1]-[#6X4] (hydrogen attached to tetravalent carbon) and [#8X2H0+0:1] (divalent oxygen with 0 hydrogens attached) atom types. The [#1:1]-[#6X4] type is exercised in all molecules in the training set, so reducing the *ε* for this type helps to reduce this overall bias. For the alkanes in the set, [#1:1]-[#6X4] is one of two parameters exercised (along with [#6X4:1], tetravalent carbon), and alkane Δ*H*_vap_ training set MSD is reduced from 4.75 kJ mol^−1^ in OpenFF 1.0.0 to an average of −0.01 kJ mol^−1^, virtually eliminating the error. Reducing the *ε* of the [#8X2H0+0:1] (ether oxygen) type helps to correct a significant overprediction of ether Δ*H*_vap_ in OpenFF 1.0.0, reducing the ether MSD from 7.95 kJ mol^−1^ to an average value of 0.54 kJ mol^−1^ after training. This reduction in error is much larger than the reduction observed after simulation-only local optimization.

The *ε*′s for [#6:1] (generic carbon) and [#8:1] (generic oxygen) present an interesting case in multidimensional optimization. We see significant changes in the *ε*′s for both [#6:1] and [#8:1]; however, the presence of more specific types in the training set means that these two types are only exercised together in a C=O double bond (a ketone, ester, or carboxylic acid). In all optimizations but run 3, we see a large reduction in [#6:1] *ε* and a slight increase in [#8:1] *ε*. The adjustment of these parameters, along with an increase in the [#6:1] *R*_min/2_, corrects an overprediction in the ester and ketone Δ*H*_vap_. Notably, simulation-only optimization against the same training set was not able to achieve the same correction for esters.

Interestingly, run 3 takes an opposite approach, increasing *ε* for [#6:1] and decreasing *ε* for [#8:1] but achieving a similar reduction in bias. This suggests that, for the purpose of this optimization, [#6:1] and [#8:1] are treated as a unit. This is not desirable in a larger context, as these parameters can appear separately in other chemical moieties, such as an alkene for [#6:1]. They are not inherently coupled and will probably lead to statistically significant errors if used in other contexts.

For *R*_min/2_, the most consistent changes are in [#6:1] and [#6X4:1], which are generally increased. Overall, the effect of increasing *R*_min/2_ should be to decrease density, as it increases inter-atomic distances and leads to higher molecular volume. This is consistent with the physical properties, as densities are slightly overpredicted in OpenFF 1.0.0, but those overpredictions are concentrated in ethers and esters. The increase in *R*_min/2_ for [#6:1] in particular helps to reduce a significant overprediction of ester densities.

#### 3.1.3 Surrogate analysis

Given that the optimizations produce diverse collection of parameter sets rather than converging on a single set, it is useful to characterize the quality of the surrogate models over the parameter space. Specifically, we compare the global accuracy of the surrogate models and measure the roughness over the surrogate models by running multiple minimizations on the final surrogate models. We performed this analysis for the *N* = 10 optimization runs.

**Fig. 10:**
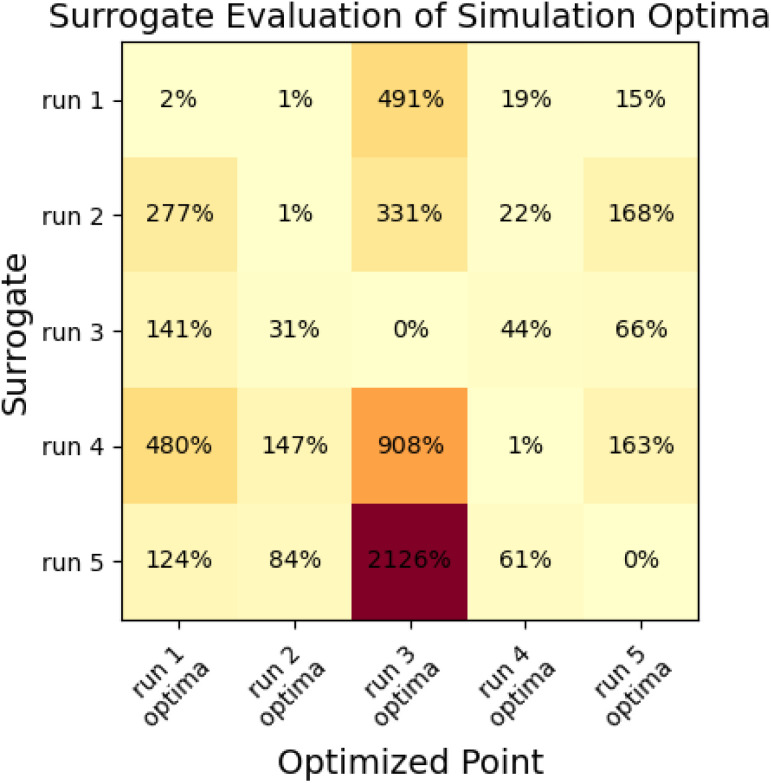
Cross-validation demonstrates that surrogates produced in the multi-fidelity optimization process are only locally predictive. Figure shows % deviation between surrogate-predicted objective functions and simulation objective functions for each of the five surrogate models and five optimization minima produced for the *N* = 10 “pure only” optimization runs.

To assess the ability of the surrogate to make accurate predictions outside the region of its minimization, we calculated objective functions with the surrogate produced in each *N* = 10 optimization for the parameter sets produced from all other *N* = 10 optimizations. If the surrogates were globally predictive, we would observe low prediction error for the other minima; with high error, surrogates are likely only locally predictive. Results are shown in Fig. 10.

This analysis indicates that surrogates produced as a part of a multi-fidelity optimization are usually only locally predictive, as many have very large prediction errors for objective functions at some of the other minima. The surrogate that performs best is the surrogate from run 1; which estimates the objective to within 20% of the simulation value for all cases besides the optima from run 3, which is far away from the region where other surrogates have samples.

We also assessed the robustness of the surrogate by performing repeated L-BFGS-B optimization on the final produced surrogates, starting from random points within the final parameter bounds box used in the optimization. This characterizes the smoothness and multimodality of the produced surrogates, as a smooth, unimodal surrogate would lead to a highly consistent local optimization, whereas a rough and multi-modal surrogate would produce different outcomes.

For the surrogate produced in each *N* = 10 multi-fideity optimization, we ran 100 L-BFGS-B optimizations from random starting points. For each of these optimizations, we calculated the standard deviation of the objective among the 100 minima (SD_*χ*_) and the percentage of optimization within 5% of the best objective (*O*_5%_). The results are shown in Table 3, along with the number of simulations used to build each surrogate (*K*_sim_).

**Table 3:** Metrics of optimization consistency for 100 L-BFGS-B optimizations starting from random points within the bounds box for the surrogates produced in each of the 5 *N* = 10 “pure-only” optimizations. SD_*χ*_ is standard deviation of resulting minimized objective functions, *O*_5%_ indicates the percentage of optimizations within 5% of the best objective for that surrogate, and *N*_sim_ indicates number of simulations used to build the optimization

Surrogate	SD_*χ*_	*O*_5%_	*K*_sim_
Optimization run 1	0.0002	99	25
Optimization run 2	0.017	63	20
Optimization run 3	0.001	72	24
Optimization run 4	0.005	94	16
Optimization run 5	0.003	49	17

The results vary widely based on the surrogate, indicating that some surrogates are more robust than others. Particularly, the surrogates from optimizations 1 and 3 have the most consistent local optimizations, even though optimization 3 produces a large parameter set outlier, with low standard deviations and ranges. These two surrogates also use the most simulation data and come from optimizations that more deeply explored their local optima, indicating that more sampling in the region of interest leads to a smoother, unimodal surrogate. Conversely, optimizations 2, 4 and 5 spend less time exploring the target region and have rougher surfaces, with L-BFGS-B optimizations less likely to converge. This indicates that exploring the target region in detail builds a more robust surrogate.

#### 3.1.4 Benchmarking

**Fig. 11:**
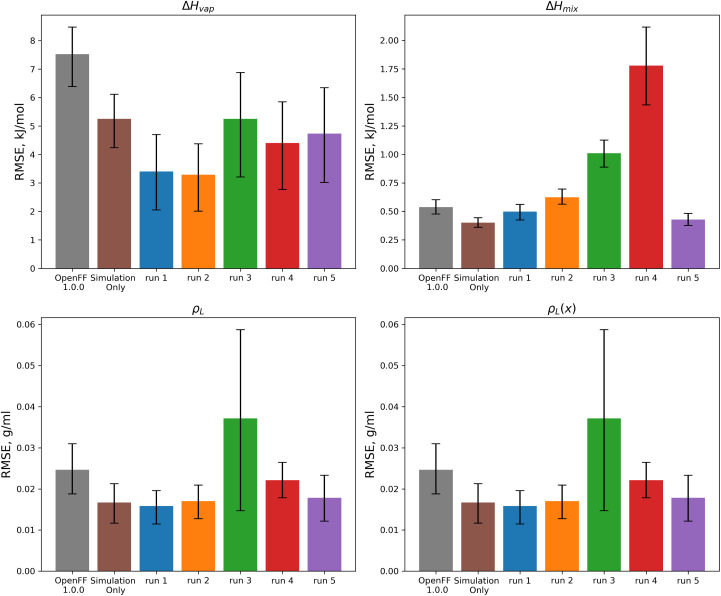
Test set RMSE for OpenFF 1.0.0, the previous simulation-only optimization, and the 5 *N* = 10 multi-fidelity runs for “pure only” targets. Benchmarking shows that some runs (such as runs 1 and 2) are transferable and outperform the simulation-only optimization, but that other runs (runs 3 and 4) have poor performance on the test set, likely due to overfitting. Error bars represent 95% confidence intervals, bootstrapped over the set of molecules in the test set.

We performed benchmarking on the test set described in Section 2.5 for OpenFF 1.0.0, the simulation-only optimization against the “pure only” set, and the 5 multi-fidelity optimization runs with *N* = 10 initial points. The benchmarking set is described in Section 2.5. We focused on the *N* = 10 runs because they generally had better objective function performance compared to the *N* = 5 runs, and they also had larger parameter changes, meaning that they would be more susceptible to overfitting. RMSE statistics for all of these force fields are plotted in Fig. 11.

These results highlight the need to test for transferability, as run 3, which had the lowest objective function over the training set, performs worse than OpenFF 1.0.0 in three of the four physical property data types in the test set. This is likely caused by the very large changes in the [#8X2H1+0:1] (hydroxyl oxygen) parameters. Similarly, run 4 performs poorly on the test set after significant changes to the [#8X2H0+0:1] *R*_min/2_ parameter.

For optimization runs without these outlier changes to parameters, such as run 1, the results are improved, with a decrease in test set Δ*H*_vap_ from an initial value in OpenFF 1.0.0 of 7.52 kJ mol^−1^ (95% CI 6.42, 8.53) to a value after fitting of 3.41 kJ mol^−1^ (95% CI 1.94, 4.68), outperforming the simulation-only optimization value of 5.25 kJ mol^−1^ (95% CI 4.31, 6.16). This improvement in Δ*H*_vap_, following the improvement in the training set, suggests that the more aggressive multi-fidelity optimization was able to adjust parameters in a way that results in better prediction of Δ*H*_vap_. For the other properties in the test set, run 1 improves over OpenFF 1.0.0 in each case, and is slightly improved over the simulation-only optimization for *ρ*_L_ and *ρ*_L_(*x*). The optimization does perform slightly worse than the simulation-only optimization for Δ*H*_mix_(*x*), which likely reflects the lack of regularization and overfitting in the surrogate model due to improved prediction of Δ*H*_vap_, which depends on vapor phase properties as well as condensed phase properties.

We also see that some of the functional-group specific reductions in the training set RMSE translate to the test set; indeed, most of the reduction in test set RMSE comes from improved treatment of ethers and esters, indicating that the parameter changes that led to these changes, such as significant reduction of ether *ε*, are transferable. Plots of the test set RMSE for all functional group categories are available in the ESI, Section 3.1.†

These results demonstrate that we can find improved parameter sets using multi-fidelity global optimization, but that care must be taken to avoid overfitting. We performed 5 optimization runs that all significantly improved the objective, but with large variations in the parameter vector solutions. There are a wide range of parameter vectors that are able to satisfy this optimization problem, but their transferability is not guaranteed. This is probably due to the training set which was chosen, as the set contains 56 target points and 12 parameters; additionally, the parameter set is “segmented” in that some parameters and targets are independent from the other parameters/targets. For example, the [#8X2H0+0:1] (ether oxygen) parameters are only dependent on the measurements of *ρ*_L_ and Δ*H*_vap_ for ethers. This leads to an optimization where overfitting is a significant concern. We could address overfitting by using a regularization scheme, as many others have done, but this many prevent us from escaping local minima in parameter space, as we hoped to do.

More physically, we can also address overfitting by broadening the training set, as more physical property targets will further constrain the optimization. In addition, including mixture data in the training set helps to guard against overfitting, given that the set becomes less segmented, as physical properties of mixtures exercise a wider range of parameters than pure physical properties.

### 3.2 Mixture training set

Implementing multi-fidelity optimization with the mixture training provides us with an opportunity to test whether we can better constrain the training data without implementing regularization, since our set of mixture data is much larger, containing 195 physical property measurements. We ran an optimization with *N* = 20 initial points, as well as one with *N* = 10 initial points, to determine what level of initial information was required to produce a successful optimization.

**Fig. 12:**
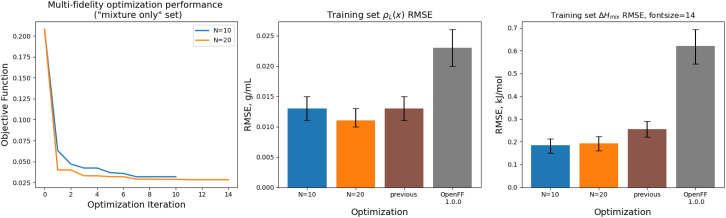
Performance of the multi-fidelity optimization algorithm on the “mixture only” training set, for runs with *N* = 10 (blue) and *N* = 20 (orange) initial parameter vectors, indicating improved performance on Δ*H*_mix_(*x*) targets when compared to simulation-only optimization. Left panel shows the objective function at each iteration of the optimization. Right two panels show the RMSE for Δ*H*_mix_(*x*) and *ρ*_L_(*x*) for the two optimizations, as well as OpenFF 1.0.0 and the previous simulation-only optimization. Error bars represent 95% confidence intervals, computed with bootstrapping over the set of molecules in the training set.

Optimization against the “mixture-only” training was successful for both the *N* = 10 and *N* = 20 runs set, achieving significant reductions in objective function and training set RMSE, as shown in Fig. 12. The *N* = 20 optimization uses a total of 34 simulation evaluations to find its optimum, whereas the *N* = 10 optimization uses only 17.

In both optimizations we observe a large drop in the objective function, followed by incremental progress until the end of the optimization. The performance is slightly improved compared to the regularized simulation-only optimization; for the *N* = 20 run the training set Δ*H*_mix_(*x*) RMSE is 0.19 kJ mol^−1^ (95% CI 0.16, 0.22) *versus* 0.24 kJ mol^−1^ (0.21, 0.29) for the simulation-only optimization. For *ρ*_L_(*x*), the RMSE is 0.011 g mL^−1^ (0.01, 0.013) *versus* 0.013 (0.011, 0.015). Both optimizations are significantly improved when compared to OpenFF 1.0.0, with Δ*H*_mix_(*x*) RMSE of 0.62 kJ mol^−1^ (0.54, 0.69) and *ρ*_L_(*x*) RMSE of 0.23 g mL^−1^ (0.02, 0.026).

**Fig. 13:**
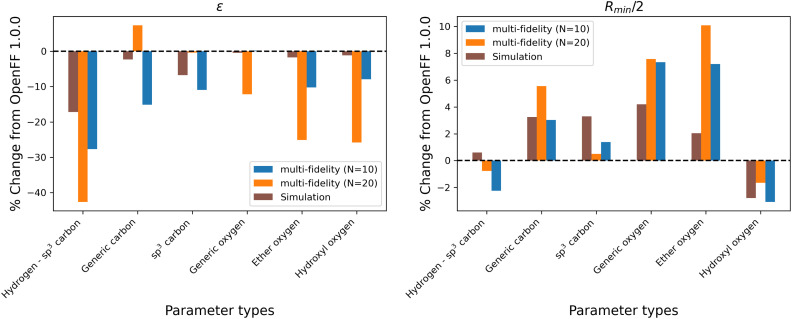
Changes in parameter values after optimization against the “mixture only” data set, relative to OpenFF 1.0.0 (the initial values), for optimizations with both *N* = 10 and *N* = 20 initial points, as well as the simulation-only solution previously obtained. Multi-fidelity optimizations show larger parameter changes, particularly in *ε*, when compared to the simulation-only optimization over the same training set, but smaller changes in parameter values when compared to the “pure-only” multi-fidelity optimizations.

While the performance is slightly improved, the parameter changes are more significant when compared to the simulation-only optimization. The changes in parameter value from OpenFF 1.0.0 are shown in Fig. 13.

Again, the changes in parameters are larger for the multi-fidelity optimizations when compared to the simulation-only optimization, particularly for the values of *ε*. However, when compared to the multi-fidelity optimization against the “pure only” training set, the changes are smaller and there are not significant outliers. We see some of the same parameter trends as in the “pure only” optimizations, like reduced values of *ε* for the [#1:1]-[#6X4] (hydrogen attached to tetravalent carbon) and [#8X2H0+0:1] (ether oxygen) atom types. A notable difference is the increased *R*_min/2_ for the [#8:1] (generic oxygen) type, which is likely related to mixture properties better capturing the hydrogen bond donor/acceptor behavior of alcohol/ester mixtures.^27^

#### 3.2.1 Benchmarking

**Fig. 14:**
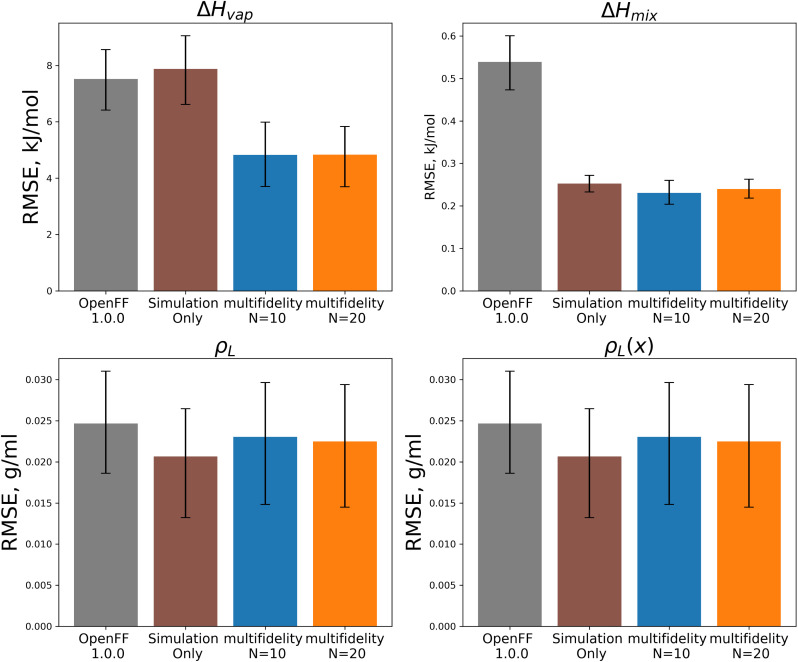
Test set RMSE for OpenFF 1.0.0, the simulation-only optimization, and both multi-fidelity optimization runs, against the “mixture only” target. Benchmarking on the test set shows the transferability of the optimized parameters; notably RMSE for both Δ*H*_mix_(*x*) and Δ*H*_vap_ are significantly improved compared to OpenFF 1.0.0, despite Δ*H*_vap_ not being included in the training, in contrast, simulation-only optimization does not improve Δ*H*_vap_. Error bars represent 95% confidence intervals, bootstrapped over the molecules in the test set.

We assessed the performance of the refit force fields on the test set. Plots of RMSEs for the four types of physical property data in the test set are shown in Fig. 14.

**Fig. 15:**
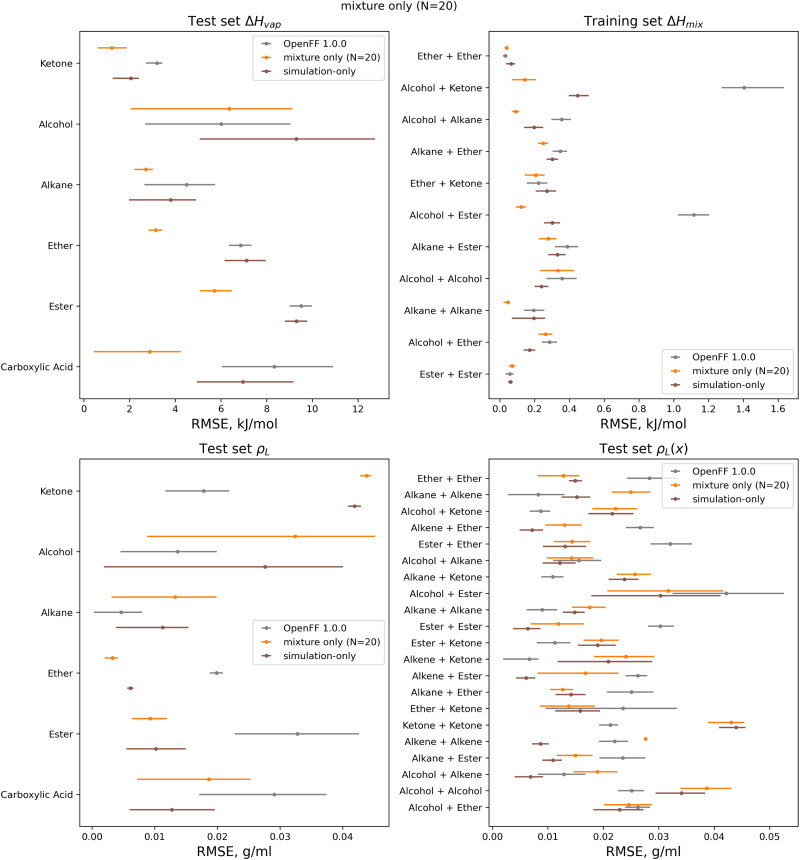
Test set RMSE for OpenFF 1.0.0, the simulation-only optimization, and both multi-fidelity runs, against the “mixture only” target and separated by function group or functional group pair. Benchmarking highlights important parameter changes and opportunities to tune the atom types in the model, including an apparent improvement of densities of ester mixtures at the expense of ketone mixture densities. Error bars represent 95% confidence intervals, bootstrapped over the molecules in the test set.

Between the simulation-only and multi-fidelity optimizations, performance is similar on pure and mixture densities (*ρ*_L_ and *ρ*_L_(*x*)), and not significantly improved when compared to OpenFF 1.0.0; densities are already well-predicted in OpenFF 1.0.0. For Δ*H*_vap_ and Δ*H*_mix_(*x*), the multi-fidelity optimizations significantly improve both properties, whereas the simulation-only optimization only improved Δ*H*_mix_(*x*). The *N* = 20 optimization has a Δ*H*_mix_ RMSE of 0.24 kJ mol^−1^ (95% CI 0.22, 0.26), similar to 0.25 (0.23, 0.27) for the simulation-only optimization. For Δ*H*_vap_, the *N* = 20 optimization has an RMSE of 4.83 kJ mol^−1^ (3.75, 5.82), significantly improved over the simulation-only optimization value of 7.87 kJ mol^−1^ (6.61, 9.14), which has slightly regressed prediction of Δ*H*_vap_ compared to the original force field. It is notable that we are able to achieve significantly improved performance on both types of enthalpy data in the test set, indicating that parameters found with the multi-fidelity optimization process achieve better transferability than a simulation-only optimization against the same data set. Examining the test set results for the *N* = 20 run separated by functional group, as shown in Fig. 15, helps to better understand how parameter changes influence force field performance.

A notable result is that esters and ketones perform better on Δ*H*_mix_(*x*) and Δ*H*_vap_ in this refit force field compared to their performance with the simulation-only refit. This may be due to changes in the parameters (bigger increases in *R*_min/2_) for the [#8:1], which is exercised only by carbonyl oxygens in both the training and test sets. However, this parameter change has an interesting effect on densities; while ester densities and densities of ester-containing mixtures are largely improved, the ketone densities regressed significantly, both with multi-fidelity optimization and simulation-only optimization. This, along with parameter gradient evidence from our previous work, suggests that splitting LJ types responsible for ketones and esters may yield improved prediction of densities.

An area where the multi-fidelity optimization struggles is in the prediction of alcohols, where outside of mixtures with the improved esters/ketones, predictions are either slightly improved or degraded. While the simulation-only optimized parameters perform slightly better on alcohols, they see similar regressions in the predictions of alcohol and alcohol-mixture densities. This may due to deficiencies in the AM1-BCC charge model for alcohols,^29^ leading to compensation in LJ parameters and reduced transferability.

Another type where splitting may yield improved results is the [#6:1] (generic carbon) type. In the training set; this type is only exercised by carbonyls, but in the test set this type is exercised by both carbonyls and alkenes. While the changes introduced in the multi-fidelity optimization significant improve performance of carbonyl-containing molecules, binary densities of alkene mixtures are significantly degraded, indicating that the type may no longer be suitable for describing both contexts. This is sensible as carbonyls and alkenes are quite different in their chemistry.

## 4 Conclusions

We present a new approach for large-scale optimization force field parameters against physical property data, based on equilibrium simulations and Gaussian process surrogate modeling. Our multi-fidelity strategy uses an iterative process of global optimization over the cheap surrogate surface and validation performed at the simulation level. We demonstrate that for reasonably sized sets of physical property data, multi-fidelity optimization can find improved parameter sets while exploring more widely than traditional local optimization techniques. Training against binary mixture data, our optimization makes larger parameter changes for ethers and carbonyls, which yield transferable improvements on test set measurements of both Δ*H*_vap_ and Δ*H*_mix_(*x*). Training against the same dataset using only local simulation-based optimization is able to achieve comparable improvements on Δ*H*_mix_(*x*), but not Δ*H*_vap_. Through examination of the training and test data, we are also able to identify targets for parameter type splitting.

While this strategy shows promise, challenges in implementation remain; one of the largest being the stochastic nature of the method. The improved parameters found are highly dependent on the set of initial parameter simulations used to build the surrogate model; the parameter space is rough and high-dimensional, meaning that Latin hypercube sampling struggles to find good starting sets of parameters. Building a better initial surrogate also requires more initial simulations, incurring higher computational expense. Analysis of the surrogates produced in the multi-fidelity process indicates that they are locally predictive models best suited to accelerating optimization, rather than global models accurate across the entire parameter space.

A potential route to improvement for this strategy is to incorporate Bayesian optimization^73^ into the parameter search strategy in order to acquire test points more efficiently. Bayesian optimization is generally efficient at solving expensive optimization problems with a limited number of objective function evaluations. Starting with a smaller and more restricted set of initial parameters and allowing Bayesian optimization to acquire samples, could lead to a more efficient and reproducible optimization.

Another target area for improvement is the robustness of the surrogate building process; roughly 50% of the optimization terminate early, as issues with automatic relevance determination (ARD) cause surrogates to sacrifice accuracy to the point where they can no longer find an improved solution. While all optimization runs still led to improved parameters overall, this suggests that parameter quality could be higher with improved surrogates. One surrogate modeling best practice that we did not incorporate into this workflow is parameter and output normalization,^51^ which can improve surrogate performance and reduce the risk of failures due to ill-conditioning, potentially yielding a more robust surrogate.

Surrogate quality could also potentially be improved by incorporating additional information from the simulations into the surrogate input. Derivative information can be obtained by reweighing^31^ and can be used to better inform the surrogate model.^74,75^ More generally, reweighting in parameter space can provide significant additional information about the surrogate model points in the local region of the simulation point.^76,77^ Reweighting in local parameter space after each simulation iteration would add relatively low computational overhead to the algorithm, compared to the cost of the simulations and optimization. Additionally, more investigation into the specifics of the optimization algorithm used over the surrogate (either the hyperparameters for the differential evolution algorithm, or other optimization algorithms entirely) could yield more efficient and consistent optimization performance.

The production of some parameter sets with drastic changes that improve training set RMSE, but are not transferable, demonstrates that overfitting is a significant risk when using more effective parameter optimization techniques. Typically, this risk is mitigated with regularization, penalizing solutions that stray too far from the initial solution. In this study, since we are interested in escaping local minima, we did not regularize the optimization; this led to the discover of some significantly improved parameters (lower values of *ε* for ether oxygens, higher values of *R*_min/2_ for carbonyl oxygens) that represented much larger changes that what regularized optimizations produced. Results from multi-fidelity optimizations on mixture properties indicate that a more complex training target can also serve to constrain an optimization and improve transferability, while allowing parameters to vary considerably. To further assess the transferability of the parameters, it would be illuminating to examine their performance on more expensive-to-compute properties, such as solvation free energies or binding free energies, as previous studies have shown that improved mixture properties give rise to improved solvation free energies^27,34^ and at least do not hurt binding free energies.^34^

This surrogate-based optimization method can be used with global optimization methods and is able to improve force field LJ parameters by escaping local minima, leading to both chemical insight and improved parameters. The success of the strategy is due to its multi-fidelity approach, using a cheaper surrogate to apply an otherwise prohibitively expensive global optimization algorithm. While already useful in its current form, the flexibility of the framework allows for significant improvement of the strategy in the future. We believe that this technique can help modelers perform better optimizations against physical property data, leading to force fields which more accurately predict the behavior of molecular systems of interest.

## Data and code availability

Software used in this paper, as well as simulated physical property datasets used to build surrogate models, are available at https://github.com/ocmadin/LJ_surrogates. To provide feedback on performance of the OpenFF force fields, we highly recommend using the issue tracker at http://github.com/openforcefield/openforcefields. For toolkit feedback, use http://github.com/openforcefield/openforcefield. Alternatively, inquiries may be e-mailed to support@openforcefield.org, though responses to e-mails sent to this address may be delayed and GitHub issues receive higher priority. For information on getting started with OpenFF, please see the documentation linked at http://github.com/openforcefield/openforcefield, and note the availability of several introductory examples. The code for surrogate model creation, optimization, data handling for this study can be found at https://github.com/ocmadin/LJ_surrogates. This repository also includes outputs of the calculations of physical properties from simulation used to build surrogate models. The version of the code employed for this study is this commit version: https://github.com/ocmadin/LJ_surrogates/commit/d7f94153801eb8c0673fe2e62c950e5beed1e999.

## Author contributions

Conceptualization: O. M., M. R. S. Methodology: O. M. Software: O. M. Investigation: O. M. Validation: O. M. Formal analysis: O. M. Data curation: O. M. Writing – original draft: O. M. and M. R. S. Writing – review & editing: O. M. and M. R. S. Visualization: O. M. Supervision: M. R. S. Project administration: M. R. S. Funding acquisition: M. R. S.

## Conflicts of interest

The authors declare the following competing financial interest(s): MRS is an Open Science Fellow with Psivant Sciences and consults for Relay Therapeutics.

## Supplementary Material

DD-002-D2DD00138A-s001
