# Supplementary material for: Using physical property surrogate models to perform accelerated multi-fidelity optimization of force field parameters
Source: Digit Discov. 2023 May 5;2(3):828–47. doi: 10.1039/d2dd00138a (PMC10259372; doi:10.1039/d2dd00138a)
Supplement: DD-002-D2DD00138A-s001 [file DD-002-D2DD00138A-s001.pdf]

Electronic Supplementary Material (ESI) for Digital Discovery.
This journal is © The Royal Society of Chemistry 2023

Supporting Information for “Using physical property surrogate models to perform accelerated multi-fidelity optimization of force field parameters”

Owen C. Madin[1,†] **and Michael R. Shirts**[1]

[1]Department of Chemical & Biological Engineering, University of Colorado Boulder, Boulder, CO, USA 80309

***For correspondence:**
michael.shirts@colorado.edu (MRS)

1 Training set composition

1.1 "Pure only"

Molecule	SMILES	Category
Acetic Acid	CC(=O)O	Acid
Methanol	CO	Alcohol
Ethanol	CCO	Alcohol
Propan-1-ol	CCCO	Alcohol
Propan-2-ol	CC(C)O	Alcohol
Butanol	CCCCO	Alcohol
2-Methylpropan-1-ol	CC(C)CO	Alcohol
2-Methylpropan-2-ol	CC(C)(C)O	Alcohol
Ethyl acetate	CCOC(C)=O	Ester
Propyl acetate	CCCOC(C)=O	Ester
Butyl acetate	CCCCOC(C)=O	Ester
Methyl formate	COC=O	Ester
Diethyl malonate	CCOC(=O)CC(=O)OCC	Ester
1,4-Dioxane	C1COCCO1	Ether
Oxane	C1CCOCC1	Ether
Methyl tert-butyl ether	COC(C)(C)C	Ether
Diisopropyl ether	CC(C)OC(C)C	Ether
Butyl ether	CCCCOCCCC	Ether
Cyclopentanone	O=C1CCCC1	Ketone
Pentan-2-one	CCCC(C)=O	Ketone
Cyclohexanone	O=C1CCCCC1	Ketone
Cycloheptanone	O=C1CCCCCC1	Ketone
Cyclohexane	C1CCCCC1	Alkane
Hexane	CCCCCC	Alkane
Methylcyclohexane	CC1CCCCC1	Alkane
Heptane	CCCCCCC	Alkane
2,2,4-Trimethylpentane	CC(C)CC(C)(C)C	Alkane
Decane	CCCCCCCCCC	Alkane

Figure 1. Molecules in the "pure only" training set, along with their corresponding SMILES strings and categories.

1.2 "Mixture only"

Molecule 1	Molecule 2	SMILES 1	SMILES 2	Category 1	Category 2
Oxane	Cyclohexanone	C1CCOCC1	O=C1CCCCC1	Ether	Ketone
Ethanol	2,2,4-Trimethylpentane	CCO	CC(C)CC(C)(C)C	Alcohol	Alkane
Butanol	Heptane	CCCCO	CCCCCCC	Alcohol	Alkane
Diisopropyl ether	2,2,4-Trimethylpentane	CC(C)OC(C)C	CC(C)CC(C)(C)C	Ether	Alkane
1,4-Dioxane	Cyclopentanone	C1COCCO1	O=C1CCCC1	Ether	Ketone
2-Methylpropan-2-ol	Butyl acetate	CC(C)(C)O	CCCCOC(C)=O	Alcohol	Ester
Propan-1-ol	Cyclohexane	CCCO	C1CCCCC1	Alcohol	Alkane
Propan-1-ol	Methylcyclohexane	CCCO	CC1CCCCC1	Alcohol	Alkane
Diethyl malonate	Butanol	CCOC(=O)CC(=O)OCC	CCCCO	Ester	Alcohol
2-Methylpropan-2-ol	Methyl formate	CC(C)(C)O	COC=O	Alcohol	Ester
Diethyl malonate	Methanol	CCOC(=O)CC(=O)OCC	CO	Ester	Alcohol
Propan-1-ol	2,2,4-Trimethylpentane	CCCO	CC(C)CC(C)(C)C	Alcohol	Alkane
Oxane	Cyclohexane	C1CCOCC1	C1CCCCC1	Ether	Alkane
Butanol	Methylcyclohexane	CCCCO	CC1CCCCC1	Alcohol	Alkane
Oxane	Pentan-2-one	C1CCOCC1	CCCC(C)=O	Ether	Ketone
1,4-Dioxane	Cyclohexanone	C1COCCO1	O=C1CCCCC1	Ether	Ketone
Butanol	2,2,4-Trimethylpentane	CCCCO	CC(C)CC(C)(C)C	Alcohol	Alkane
1,4-Dioxane	Pentan-2-one	C1COCCO1	CCCC(C)=O	Ether	Ketone
Butanol	Hexane	CCCCO	CCCCCC	Alcohol	Alkane
Oxane	Methylcyclohexane	C1CCOCC1	CCCCCCC	Ether	Alkane
Diethyl malonate	2-Methylpropan-1-ol	CCOC(=O)CC(=O)OCC	CC(C)CO	Ester	Alcohol
Ethanol	Ethyl acetate	CCO	CCOC(C)=O	Alcohol	Ester
Diethyl malonate	Propan-2-ol	CCOC(=O)CC(=O)OCC	CC(C)O	Ester	Alcohol
1,4-Dioxane	Cycloheptanone	C1COCCO1	O=C1CCCCCC1	Ether	Ketone
Methyl formate	Methanol	COC=O	CO	Ester	Alcohol
Methyl tert-butyl ether		COC(C)(C)C	CCCCCCCCCC	Ether	Alkane
Oxane	Hexane	C1CCOCC1	CCCCCC	Ether	Alkane
Diisopropyl ether	Heptane	CC(C)OC(C)C	CCCCCCC	Ether	Alkane
Diethyl malonate	Ethanol	CCOC(=O)CC(=O)OCC	CCO	Ester	Alcohol
Ethanol	Acetic Acid	CCO	CC(=O)O	Alcohol	Acid
Oxane	Cyclopentanone	C1CCOCC1	O=C1CCCC1	Ether	Ketone
Butyl ether	2,2,4-Trimethylpentane	CCCCOCCCC	CC(C)CC(C)(C)C	Ether	Alkane

Figure 2. Molecules in the binary mixtures in the "mixture only" training set, along with their corresponding SMILES strings and categories.

2 Training set performance

2.1 "Pure only" optimization, N=5

2.1.1 Run 1

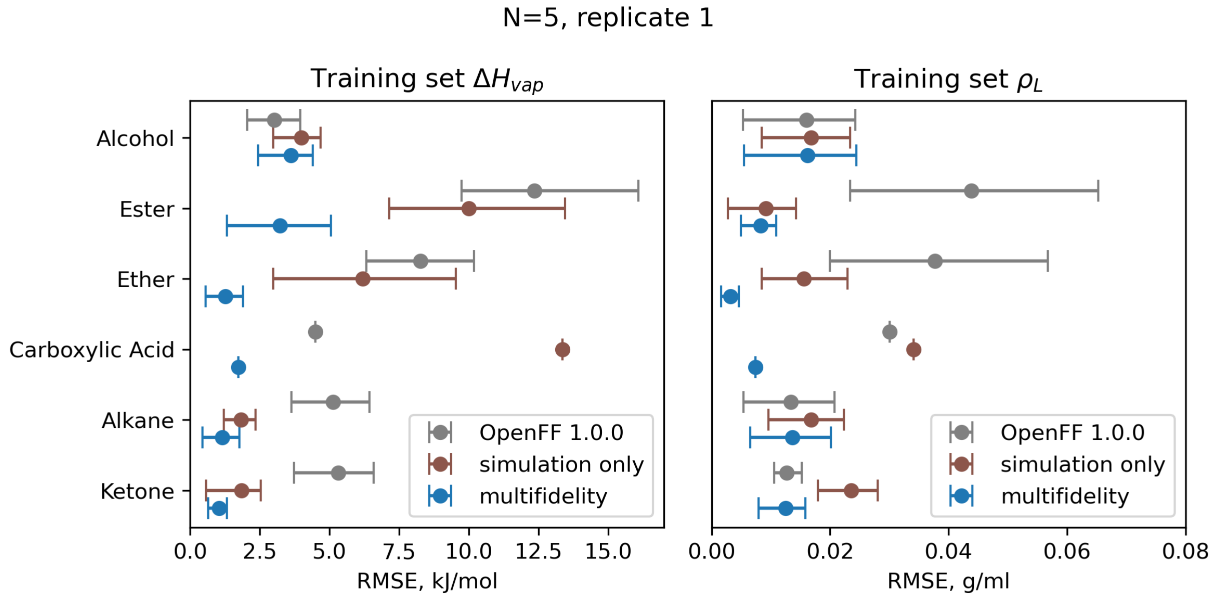

Figure 3. RMSE in training set ΔH_{vap} and ρ_L, for OpenFF 1.0.0, simulation-only refit parameters, and retrained parameters from N=5 initial points multi-fidelity run 1. Error bars represent bootstrapped 95% confidence intervals

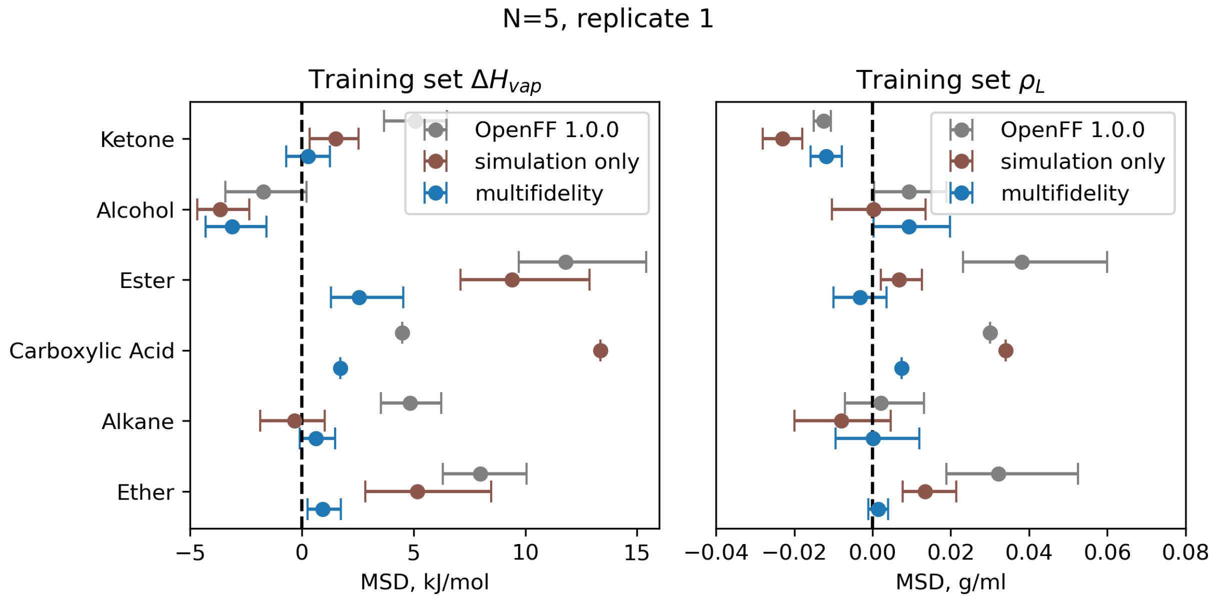

Figure 4. Bias in training set ΔH_{vap} and ρ_L, as measured by the mean signed deviation (MSD), for OpenFF 1.0.0, simulation-only refit, and retrained parameters from N=5 multi-fidelity run 1. Error bars represent bootstrapped 95% confidence intervals

2.1.2 Run 2

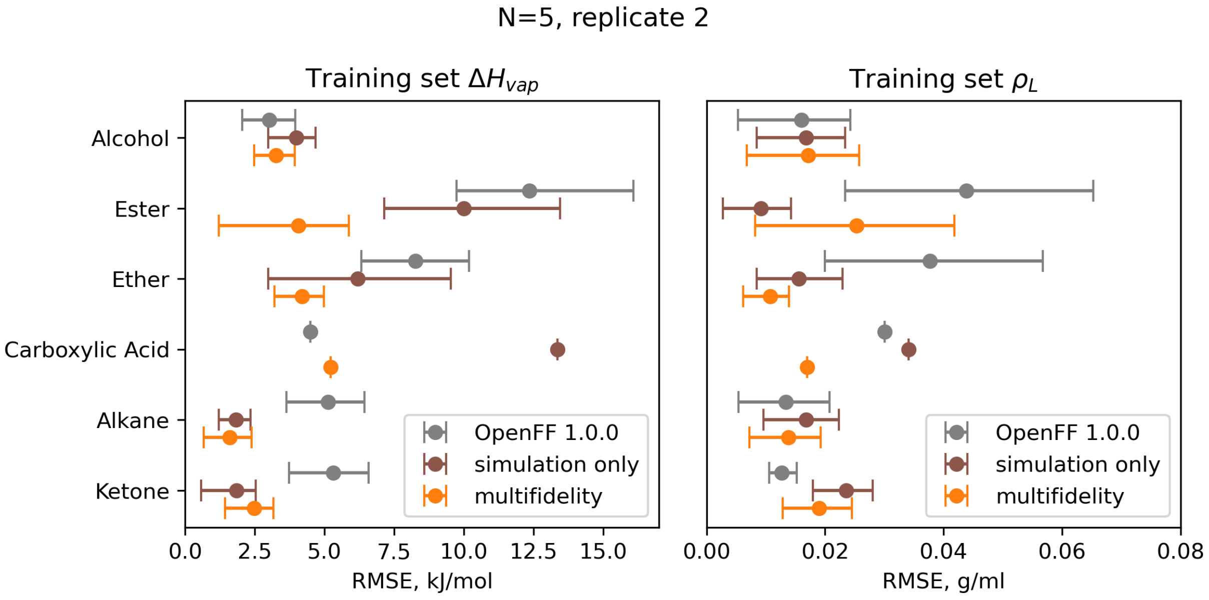

Figure 5. RMSE in training set ΔH_{vap} and ρ_L, for OpenFF 1.0.0, simulation-only refit parameters, and retrained parameters from N=5 initial points multi-fidelity run 2. Error bars represent bootstrapped 95% confidence intervals

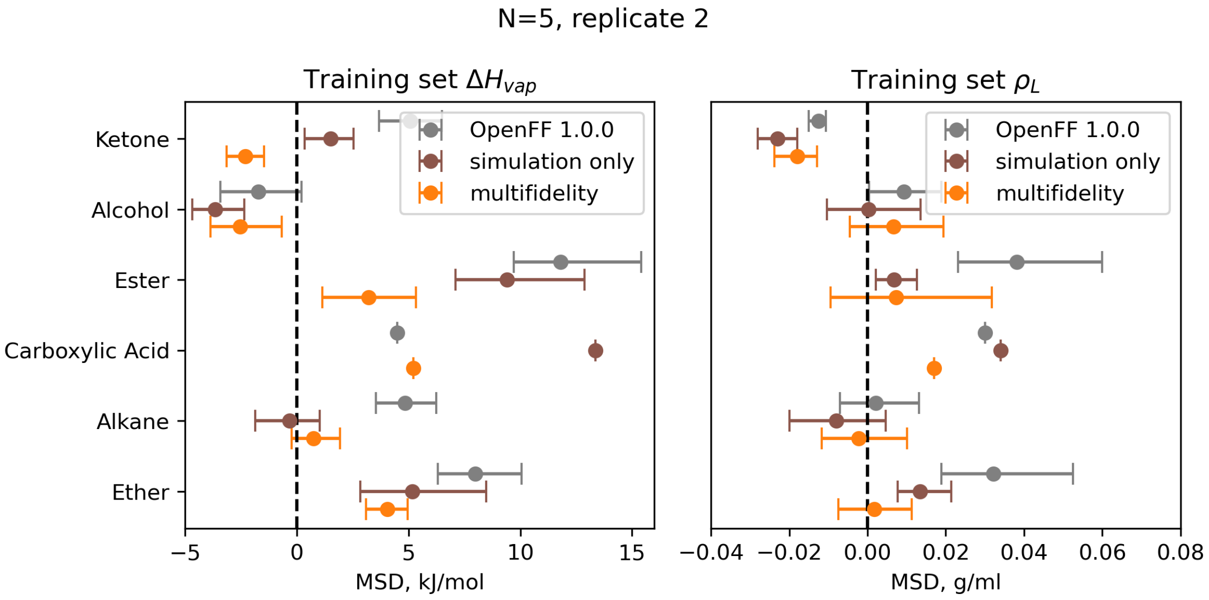

Figure 6. Bias in training set ΔH_{vap} and ρ_L, as measured by the mean signed deviation (MSD), for OpenFF 1.0.0, simulation-only refit, and retrained parameters from N=5 multi-fidelity run 2. Error bars represent bootstrapped 95% confidence intervals

2.1.3 Run 3

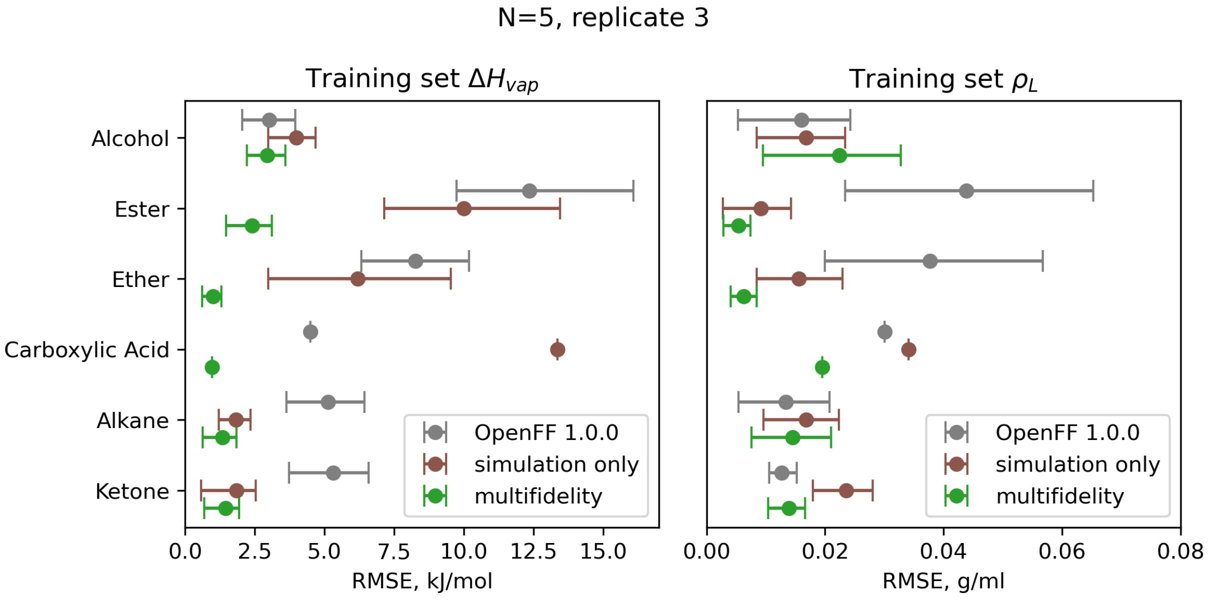

Figure 7. RMSE in training set ΔH_{vap} and ρ_L, for OpenFF 1.0.0, simulation-only refit parameters, and retrained parameters from N=5 initial points multi-fidelity run 3. Error bars represent bootstrapped 95% confidence intervals

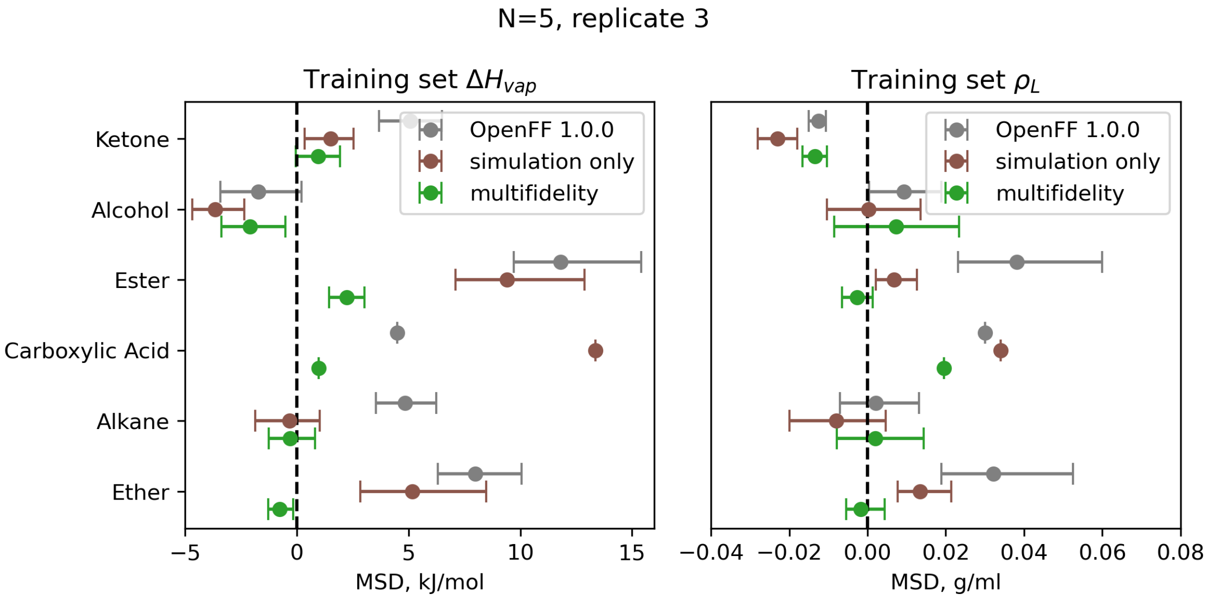

Figure 8. Bias in training set ΔH_{vap} and ρ_L, as measured by the mean signed deviation (MSD), for OpenFF 1.0.0, simulation-only refit, and retrained parameters from N=5 multi-fidelity run 3. Error bars represent bootstrapped 95% confidence intervals

2.1.4 Run 4

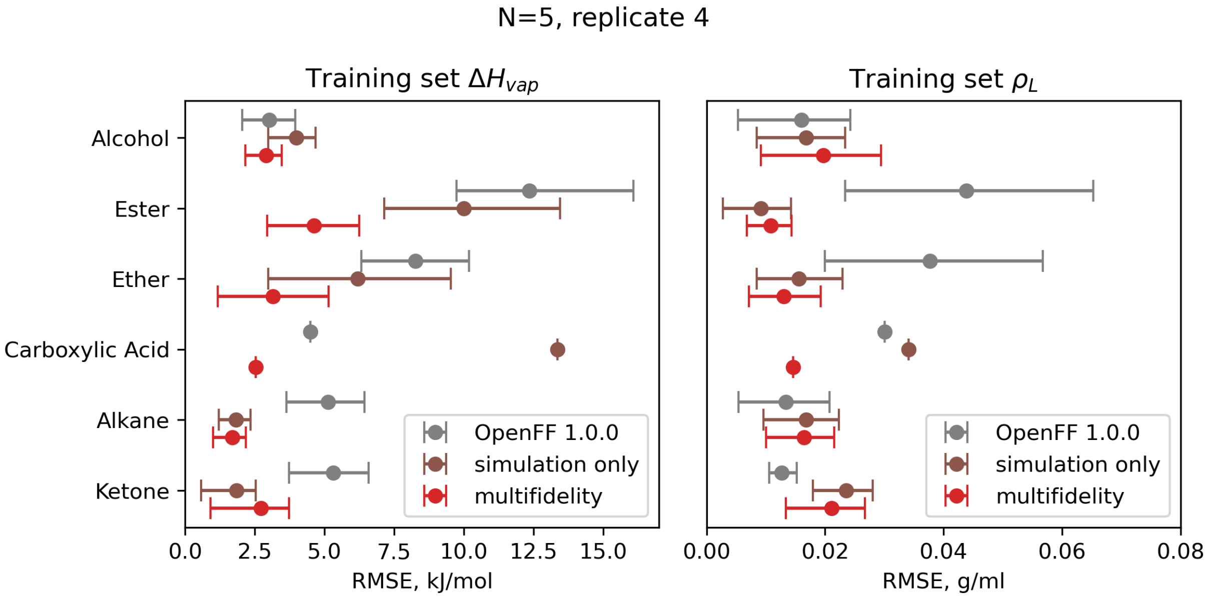

Figure 9. RMSE in training set ΔH_{vap} and ρ_L, for OpenFF 1.0.0, simulation-only refit parameters, and retrained parameters from N=5 initial points multi-fidelity run 4. Error bars represent bootstrapped 95% confidence intervals

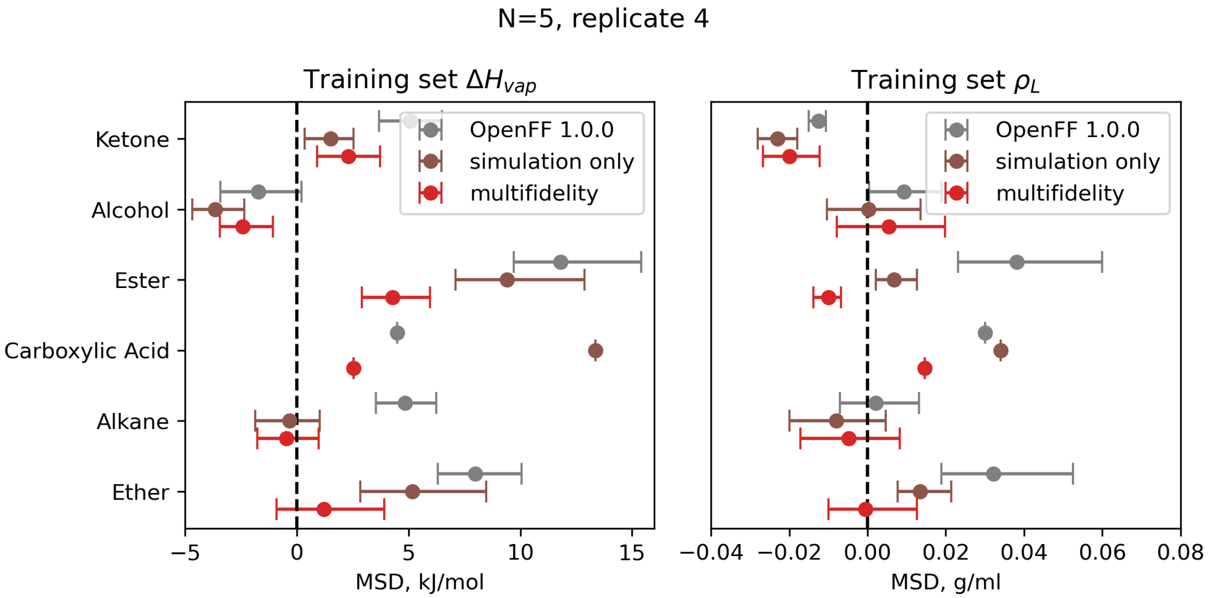

Figure 10. Bias in training set ΔH_{vap} and ρ_L, as measured by the mean signed deviation (MSD), for OpenFF 1.0.0, simulation-only refit, and retrained parameters from N=5 multi-fidelity run 4. Error bars represent bootstrapped 95% confidence intervals

2.1.5 Run 5

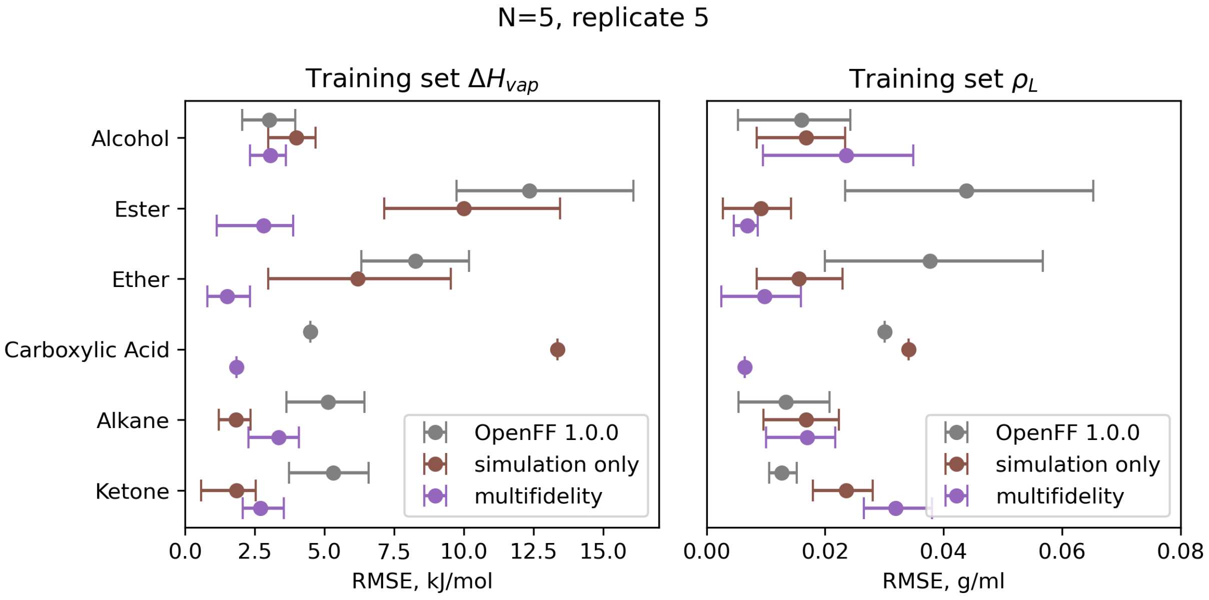

Figure 11. RMSE in training set ΔH_{vap} and ρ_L, for OpenFF 1.0.0, simulation-only refit parameters, and retrained parameters from N=5 initial points multi-fidelity run 5. Error bars represent bootstrapped 95% confidence intervals

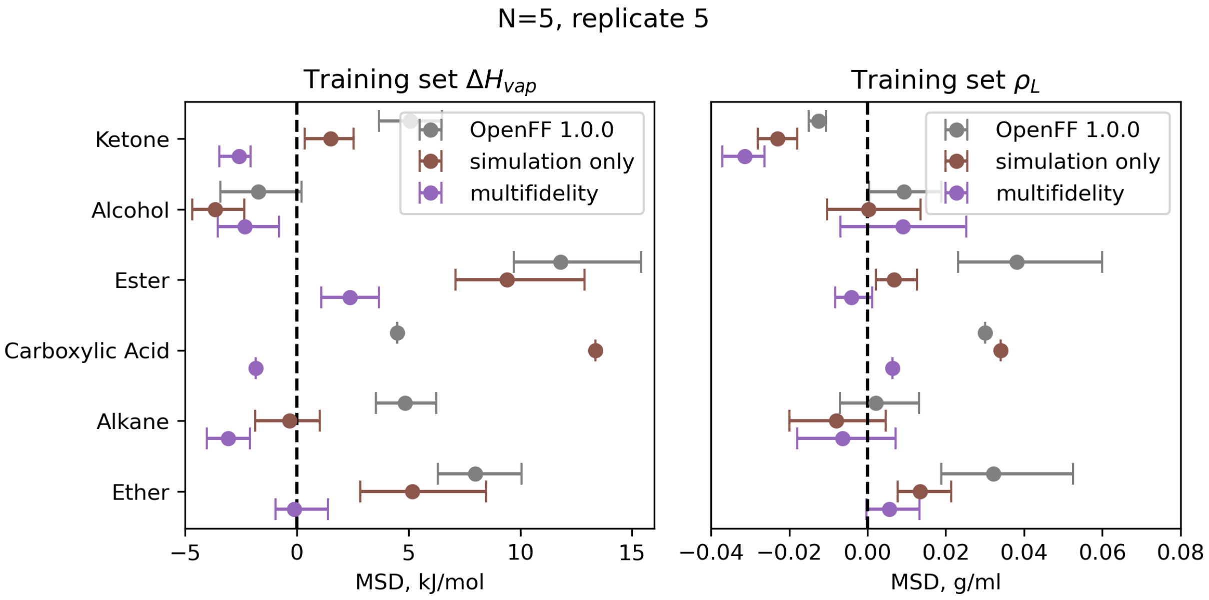

Figure 12. Bias in training set ΔH_{vap} and ρ_L, as measured by the mean signed deviation (MSD), for OpenFF 1.0.0, simulation-only refit, and retrained parameters from N=5 multi-fidelity run 5. Error bars represent bootstrapped 95% confidence intervals

2.2 “Pure only” optimization, N=10

2.2.1 Run 1

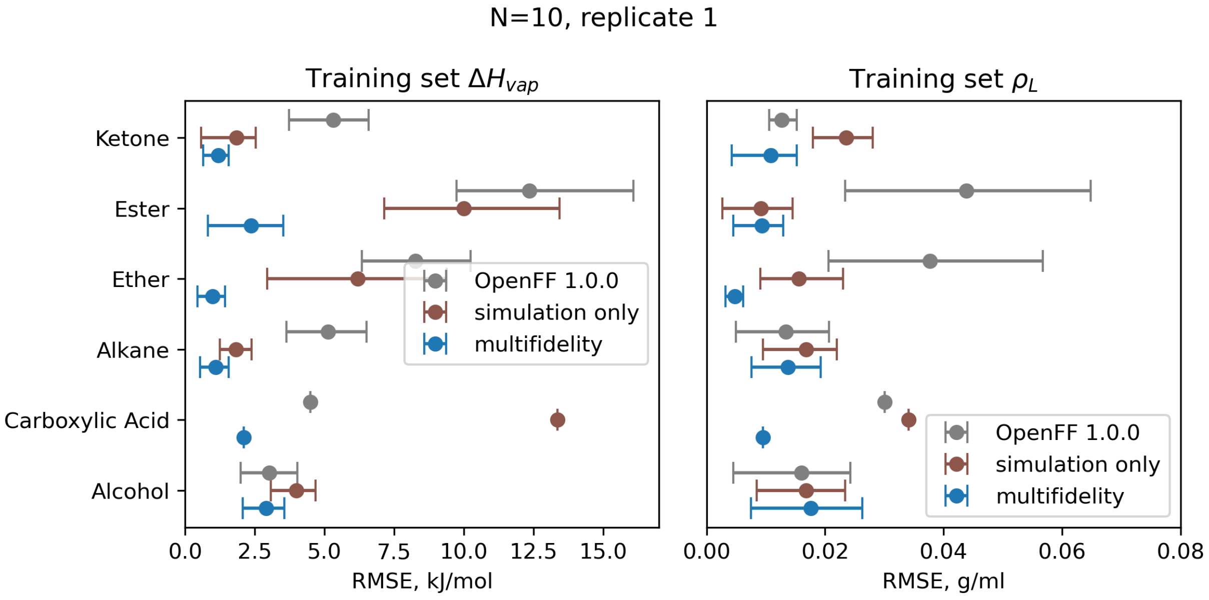

Figure 13. RMSE in training set ΔH_{vap} and ρ_L, for OpenFF 1.0.0, simulation-only refit parameters, and retrained parameters from N=10 initial points multi-fidelity run 1. Error bars represent bootstrapped 95% confidence intervals

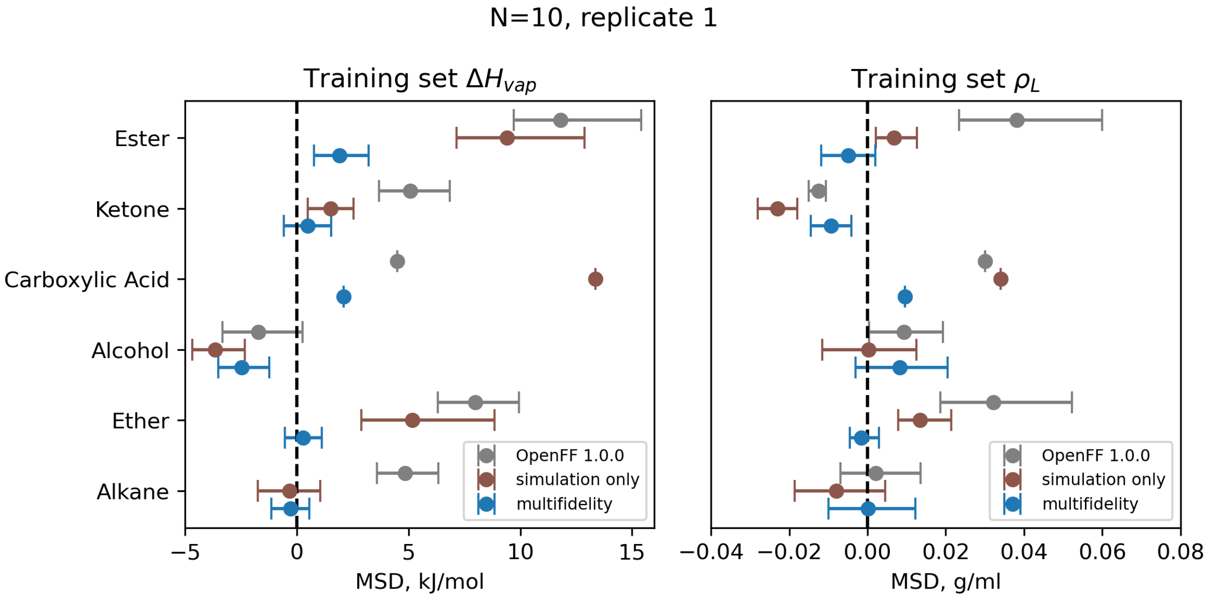

Figure 14. Bias in training set ΔH_{vap} and ρ_L, as measured by the mean signed deviation (MSD), for OpenFF 1.0.0, simulation-only refit, and retrained parameters from N=10 multi-fidelity run 1. Error bars represent bootstrapped 95% confidence intervals

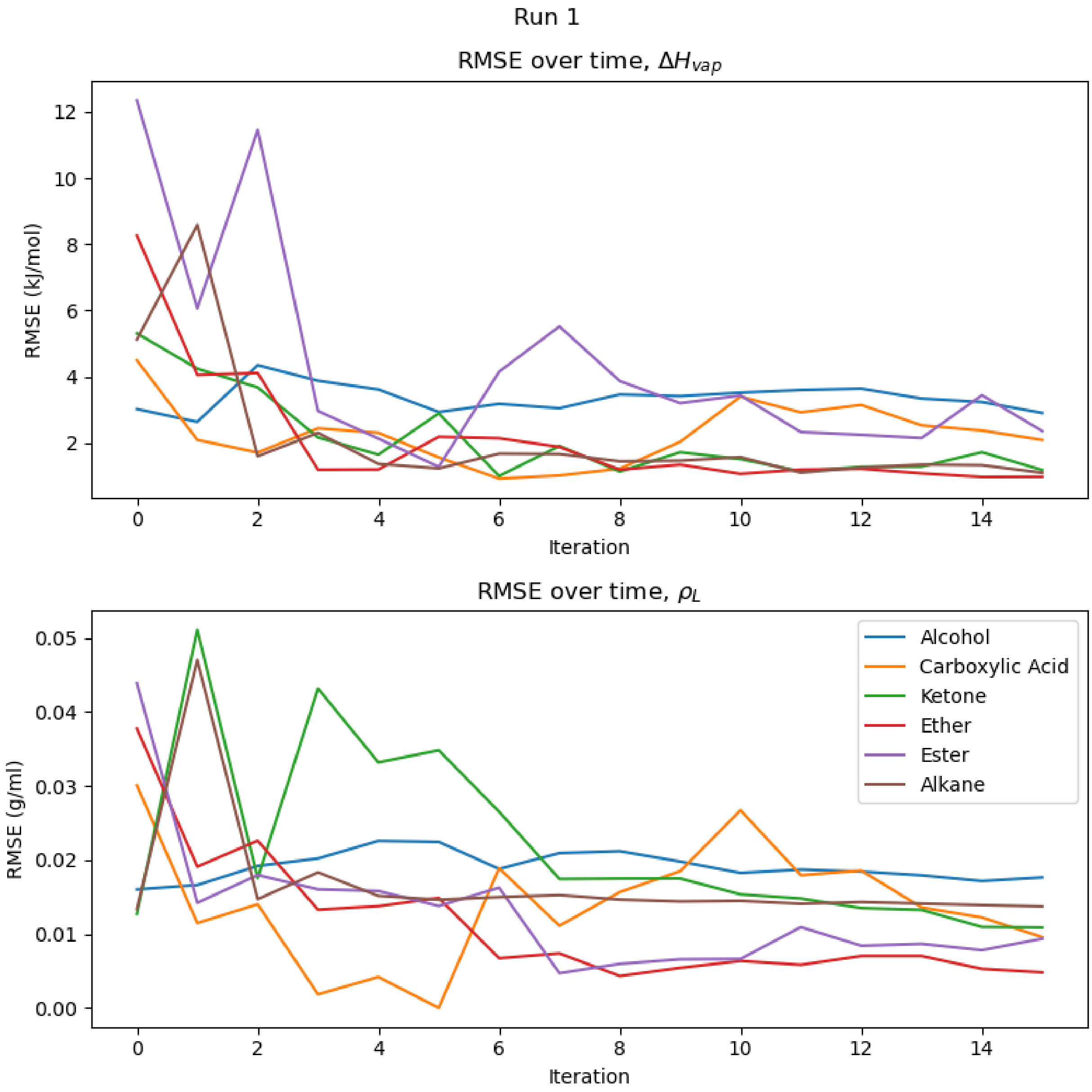

Figure 15. Per-moiety training set RMSE at each accepted optimization step, for N=10 multi-fidelity run 1.

2.2.2 Run 2

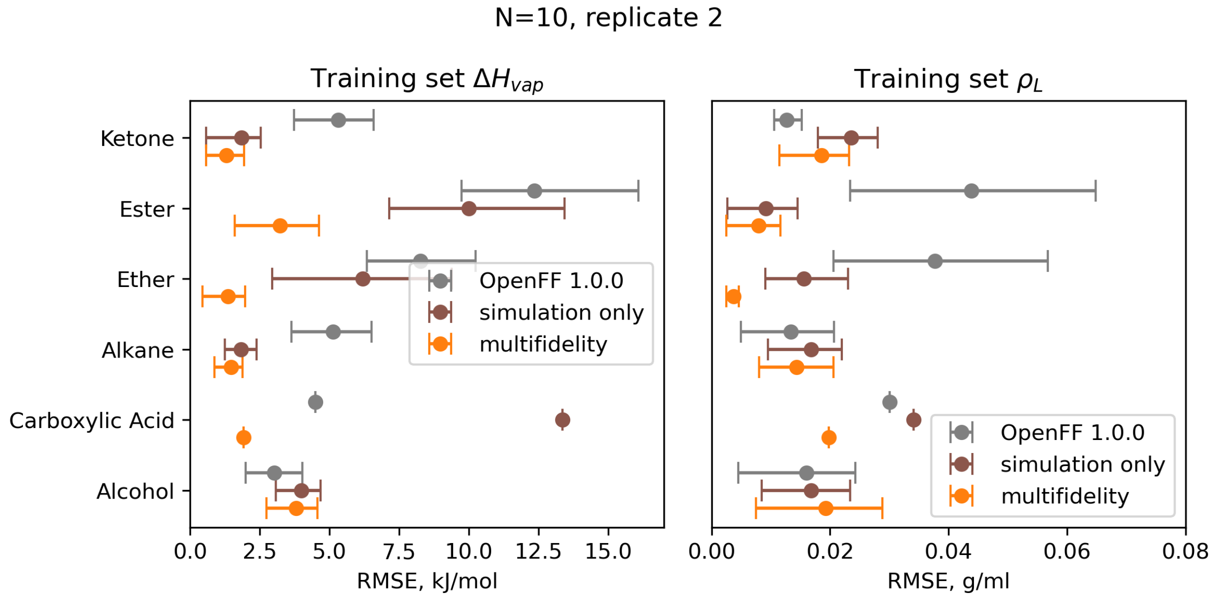

Figure 16. RMSE in training set ΔH_{vap} and ρ_L, for OpenFF 1.0.0, simulation-only refit parameters, and retrained parameters from N=10 initial points multi-fidelity run 2. Error bars represent bootstrapped 95% confidence intervals

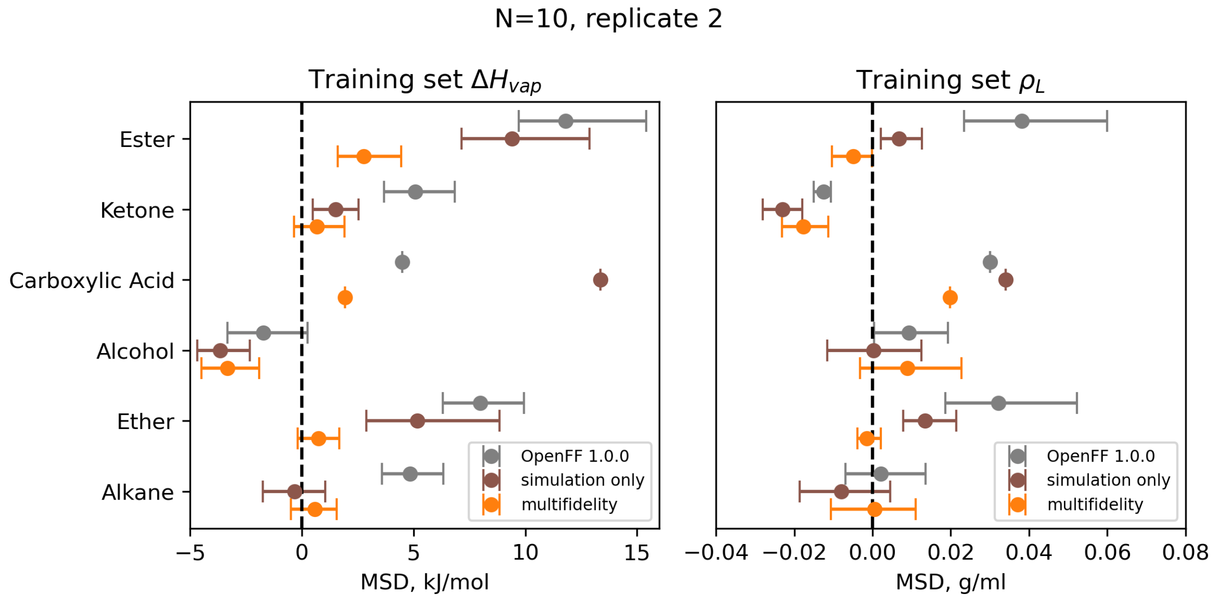

Figure 17. Bias in training set ΔH_{vap} and ρ_L, as measured by the mean signed deviation (MSD), for OpenFF 1.0.0, simulation-only refit, and retrained parameters from N=10 multi-fidelity run 2. Error bars represent bootstrapped 95% confidence intervals

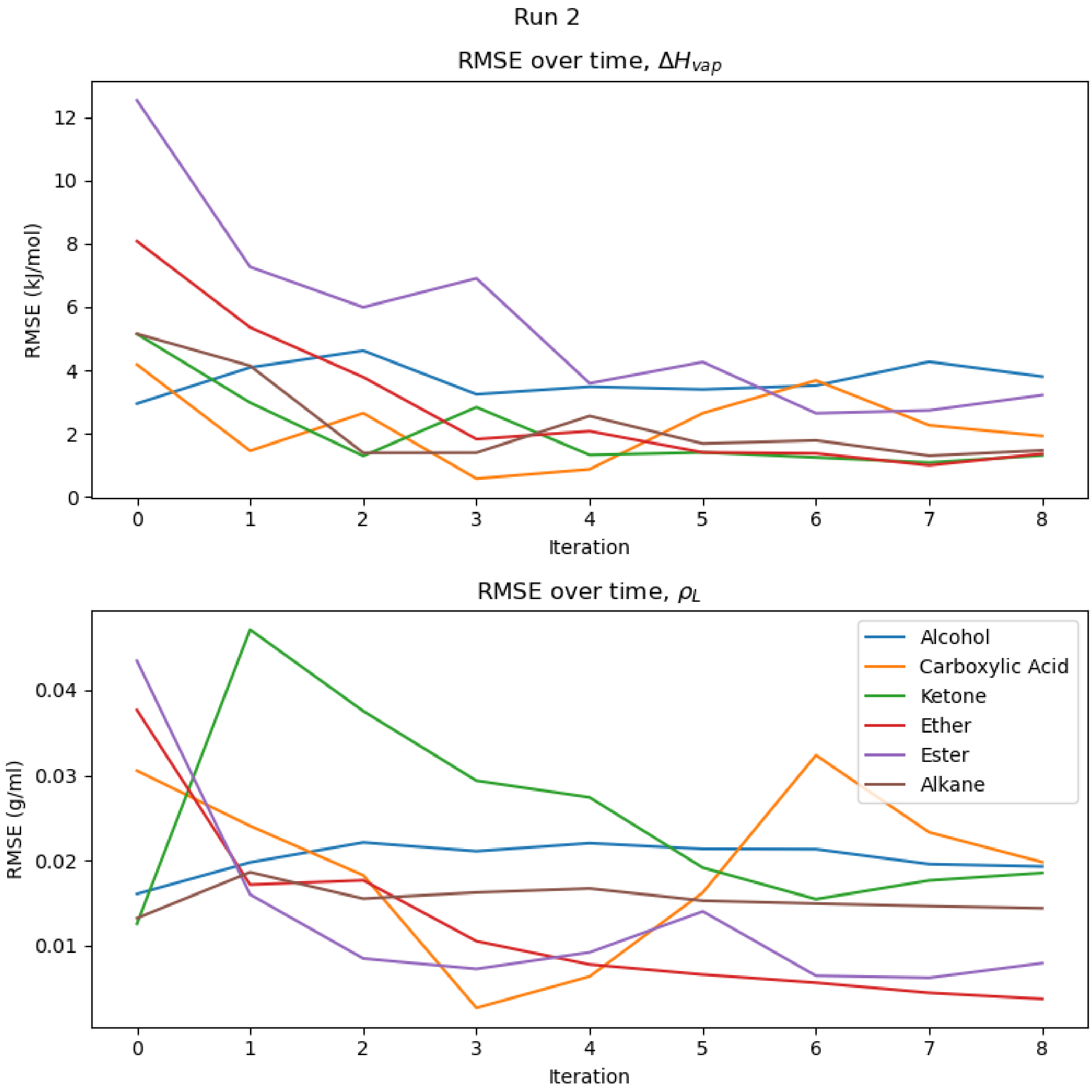

Figure 18. Per-moiety training set RMSE at each accepted optimization step, for N=10 multi-fidelity run 2.

2.2.3 Run 3

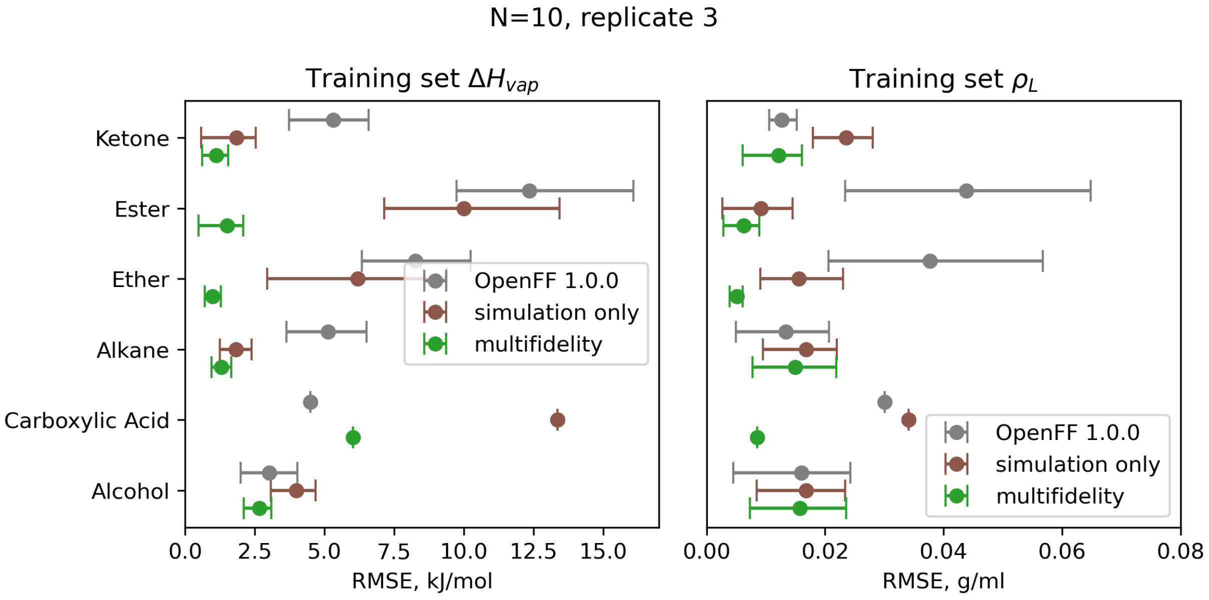

Figure 19. RMSE in training set ΔH_{vap} and ρ_L, for OpenFF 1.0.0, simulation-only refit parameters, and retrained parameters from N=10 initial points multi-fidelity run 3. Error bars represent bootstrapped 95% confidence intervals

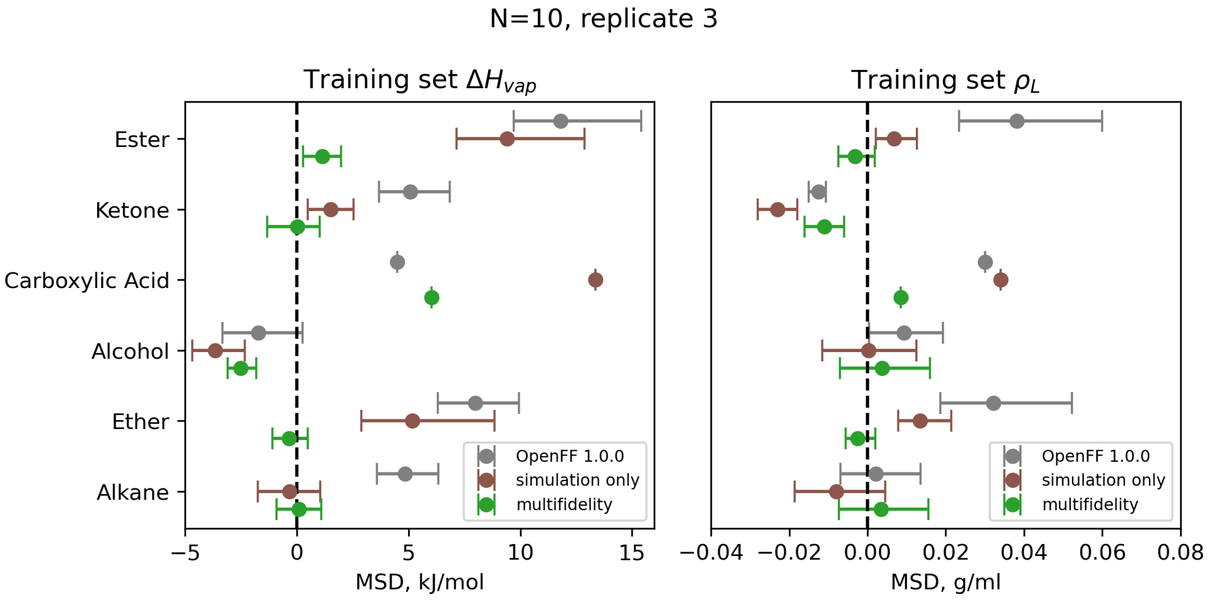

Figure 20. Bias in training set ΔH_{vap} and ρ_L, as measured by the mean signed deviation (MSD), for OpenFF 1.0.0, simulation-only refit, and retrained parameters from N=10 multi-fidelity run 3. Error bars represent bootstrapped 95% confidence intervals

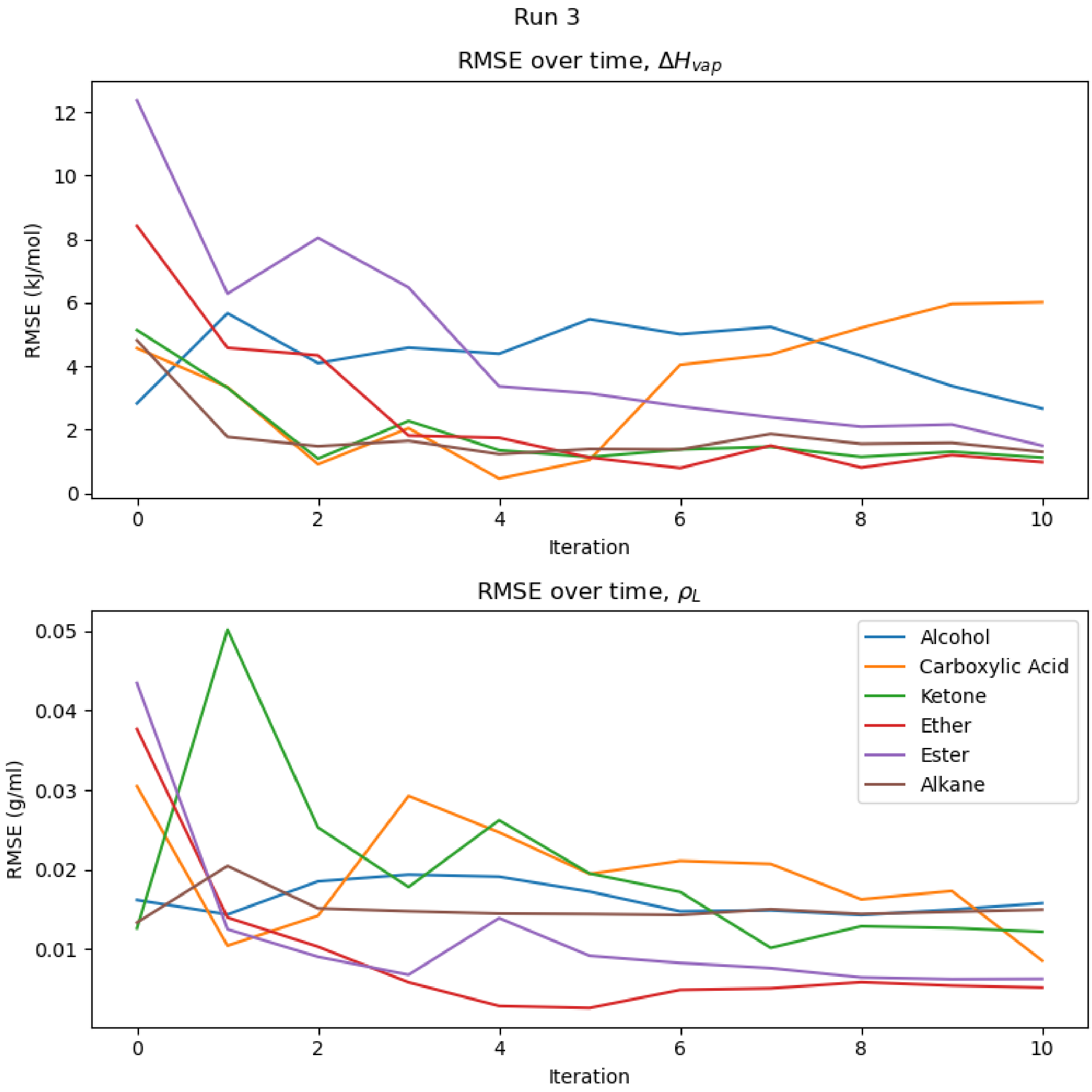

Figure 21. Per-moiety training set RMSE at each accepted optimization step, for N=10 multi-fidelity run 3.

2.2.4 Run 4

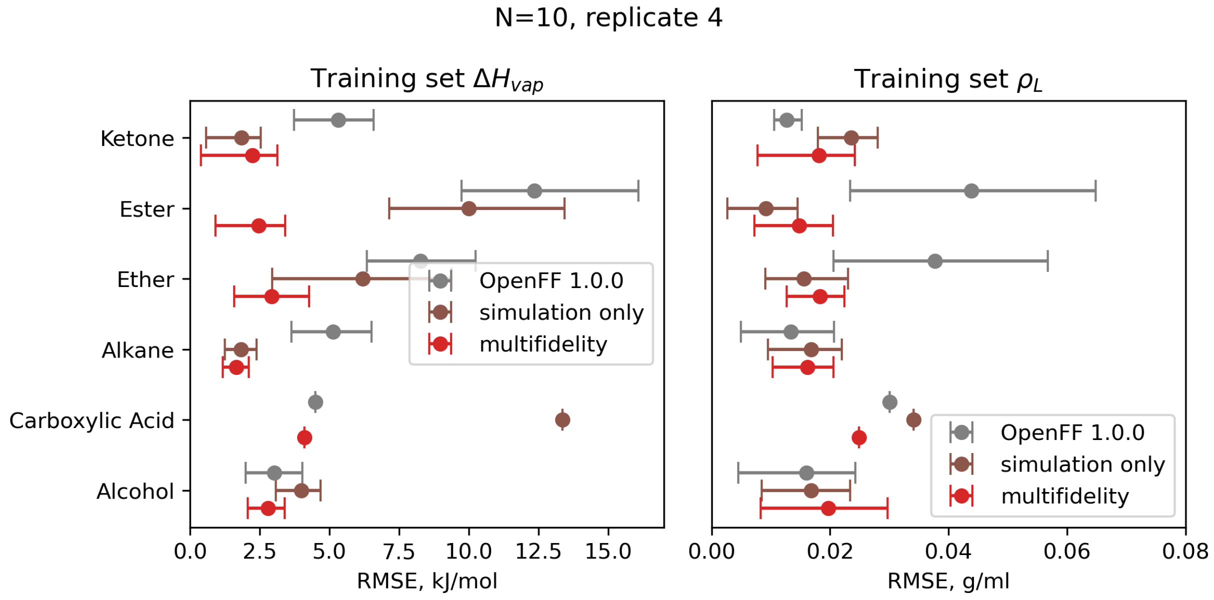

Figure 22. RMSE in training set ΔH_{vap} and ρ_L, for OpenFF 1.0.0, simulation-only refit parameters, and retrained parameters from N=10 initial points multi-fidelity run 4. Error bars represent bootstrapped 95% confidence intervals

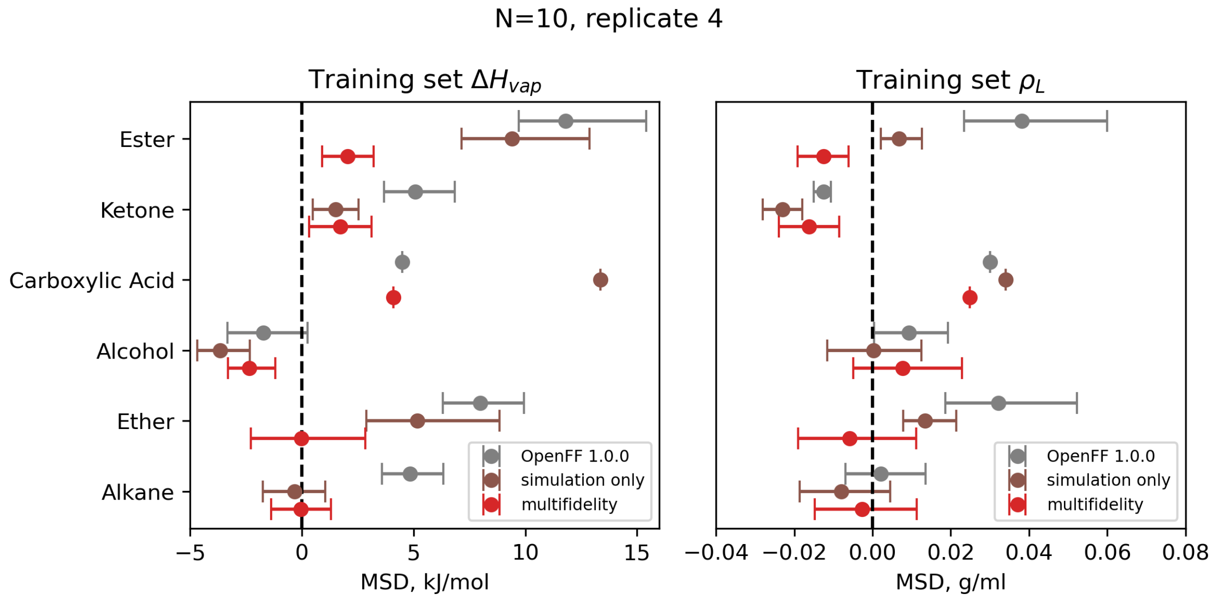

Figure 23. Bias in training set ΔH_{vap} and ρ_L, as measured by the mean signed deviation (MSD), for OpenFF 1.0.0, simulation-only refit, and retrained parameters from N=10 multi-fidelity run 4. Error bars represent bootstrapped 95% confidence intervals

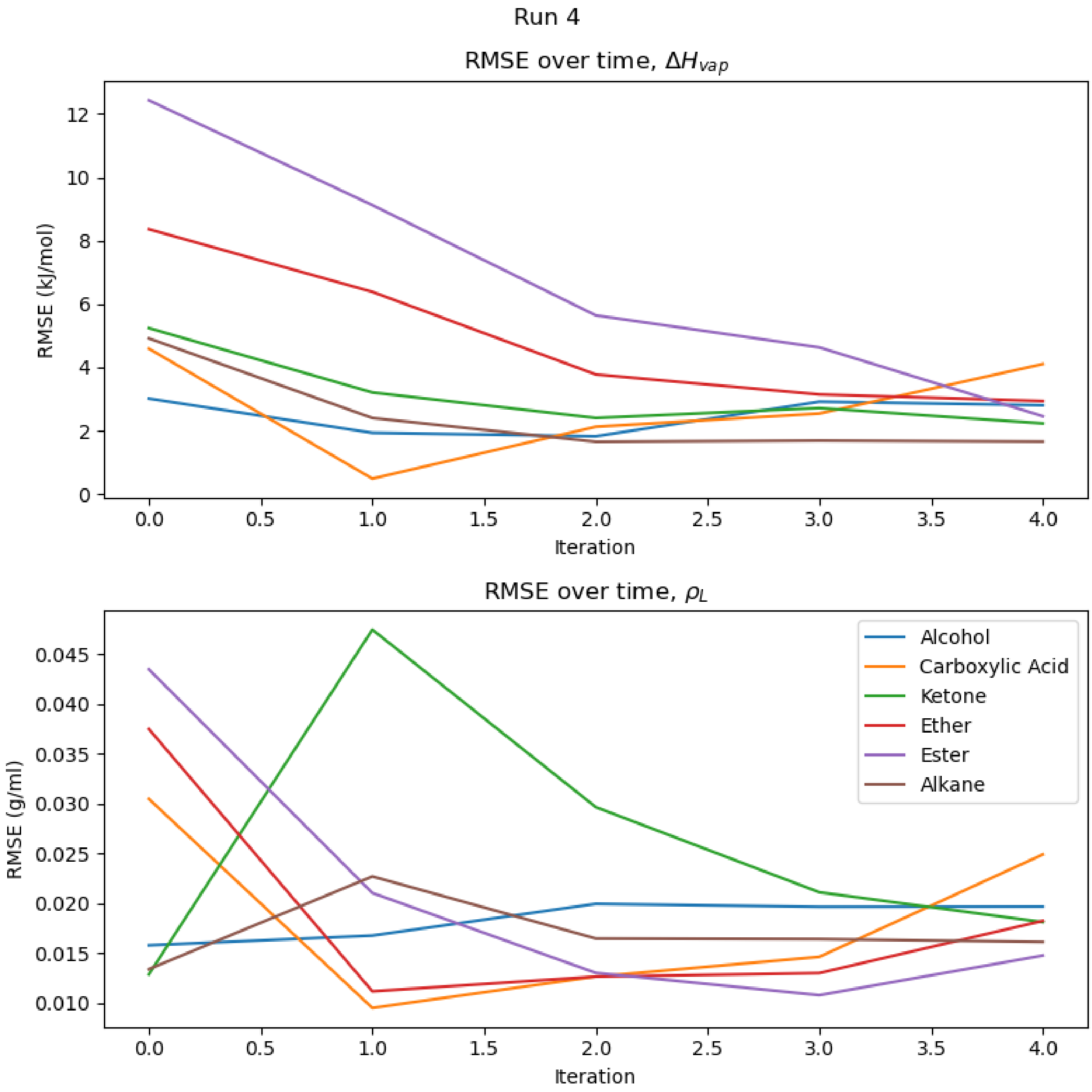

Figure 24. Per-moiety training set RMSE at each accepted optimization step, for N=10 multi-fidelity run 4.

2.2.5 Run 5

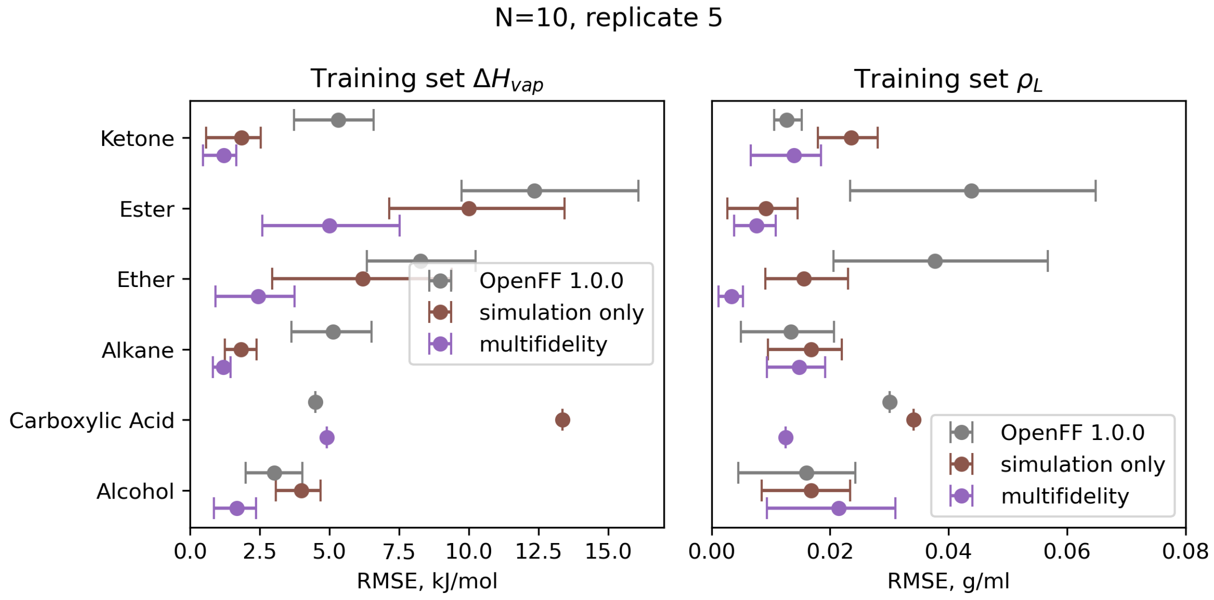

Figure 25. RMSE in training set ΔH_{vap} and ρ_L, for OpenFF 1.0.0, simulation-only refit parameters, and retrained parameters from N=10 initial points multi-fidelity run 5. Error bars represent bootstrapped 95% confidence intervals

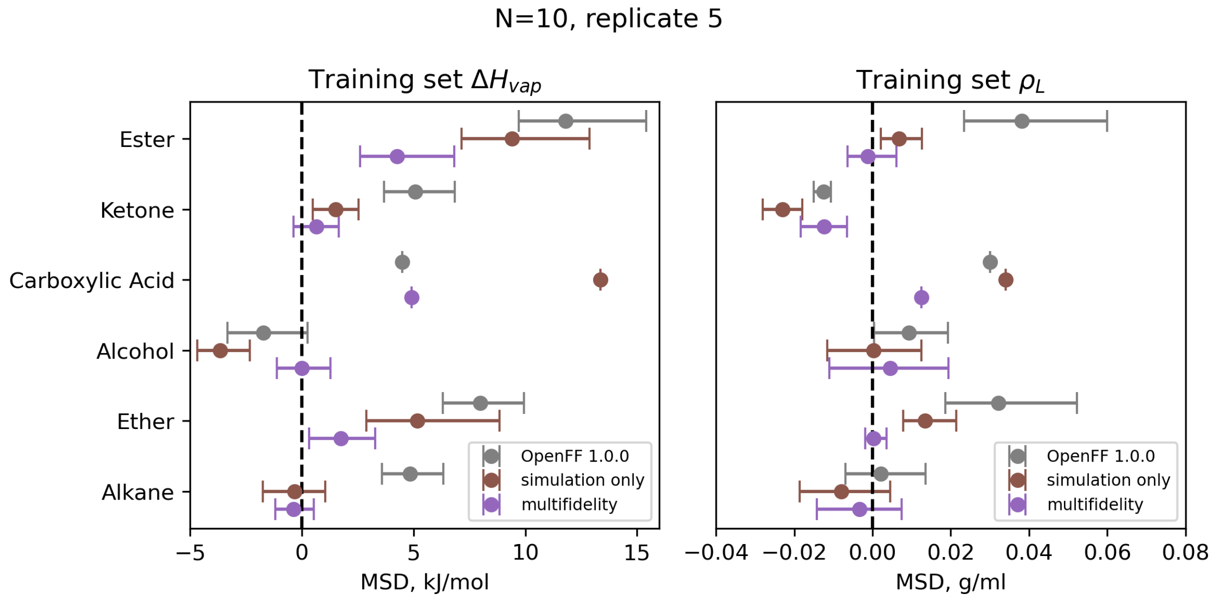

Figure 26. Bias in training set ΔH_{vap} and ρ_L, as measured by the mean signed deviation (MSD), for OpenFF 1.0.0, simulation-only refit, and retrained parameters from N=10 multi-fidelity run 5. Error bars represent bootstrapped 95% confidence intervals

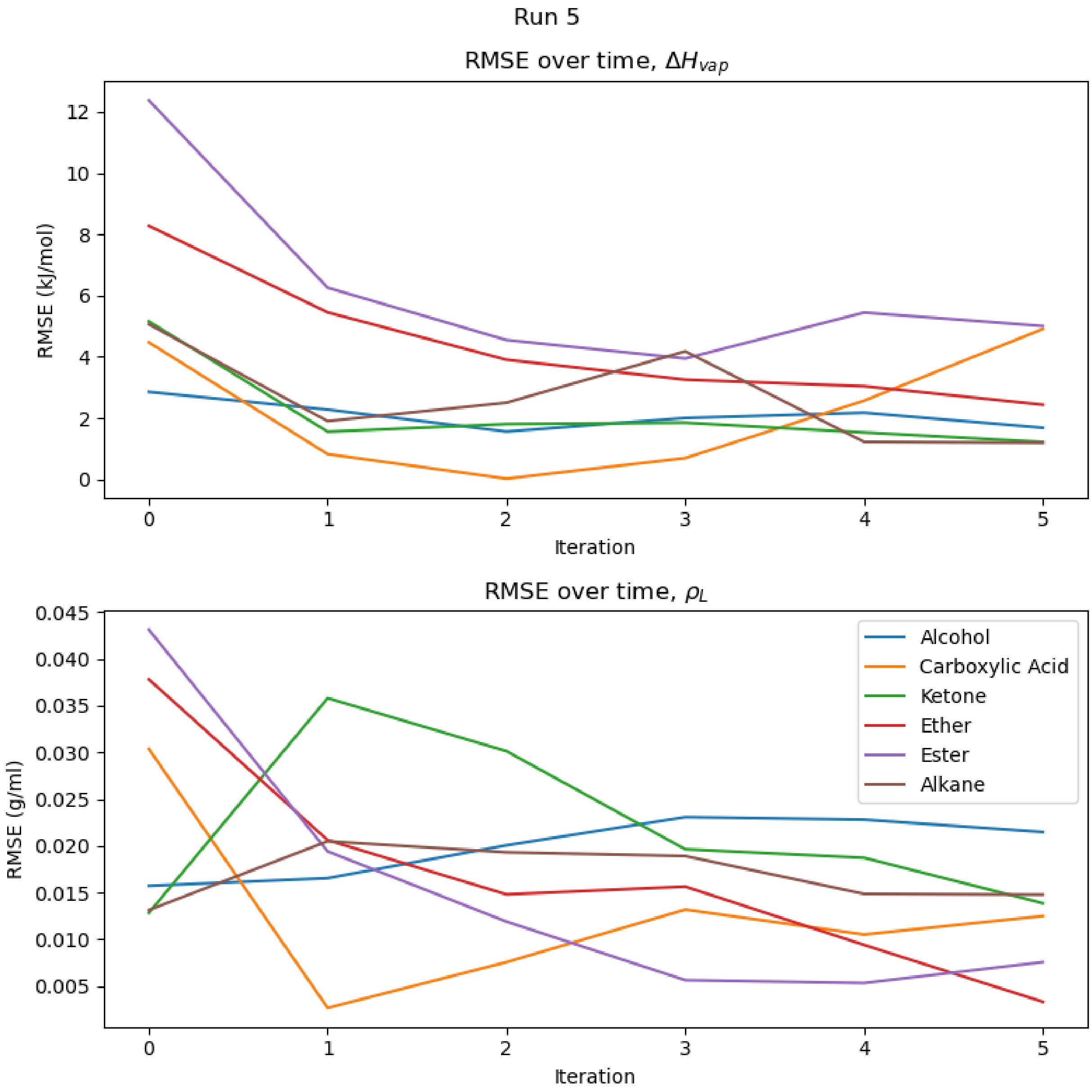

Figure 27. Per-moiety training set RMSE at each accepted optimization step, for N=10 multi-fidelity run 5.

2.3 "Mixture only" optimization, N=10

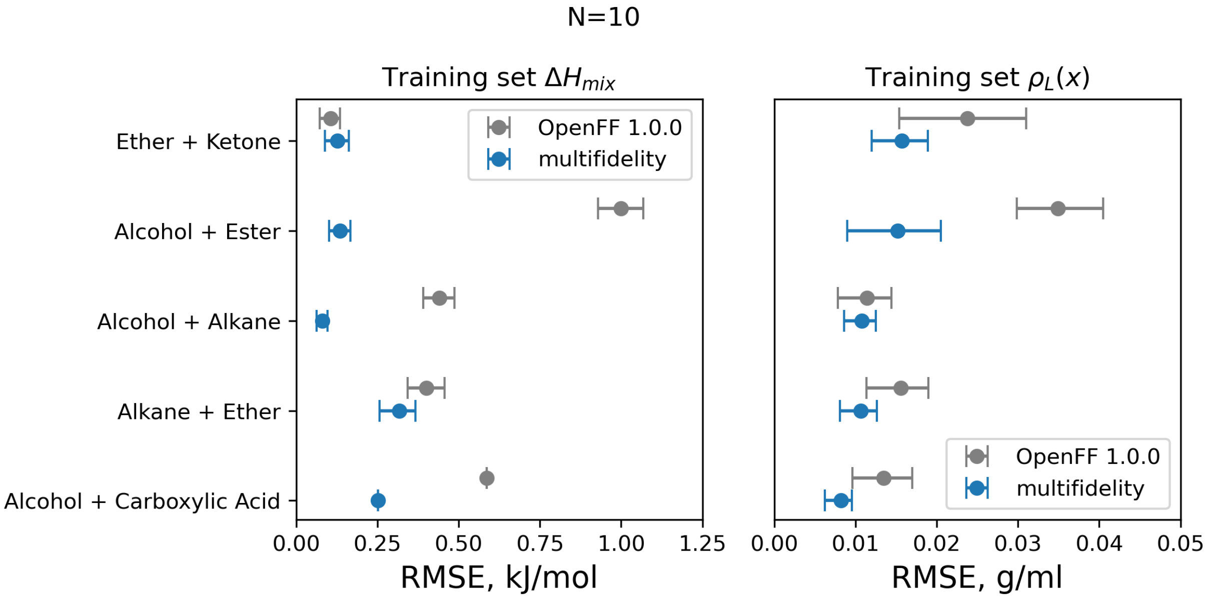

Figure 28. RMSE in training set ΔH_{mix} and $\rho_L(x)$, for OpenFF 1.0.0 and retrained parameters from N=10 initial points multi-fidelity optimization against "mixture-only" training set. Error bars represent bootstrapped 95% confidence intervals

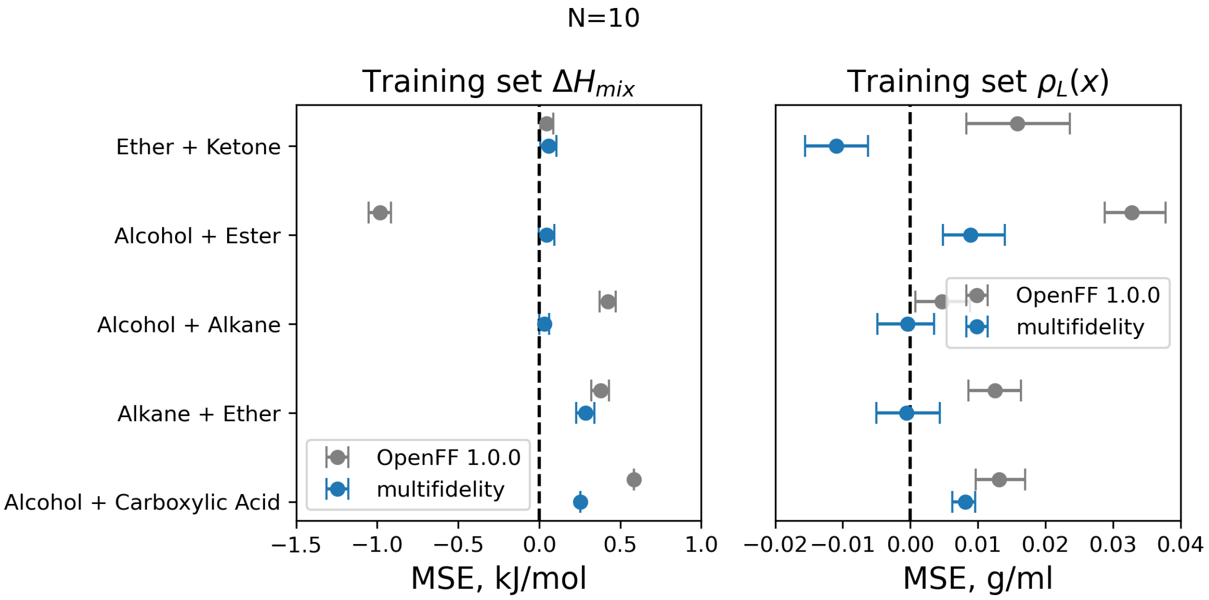

Figure 29. Bias in training set ΔH_{mix} and $\rho_L(x)$, as measured by the mean signed deviation (MSD), for OpenFF 1.0.0 and retrained parameters from N=10 multi-fidelity optimization against "mixture-only" training set. Error bars represent bootstrapped 95% confidence intervals

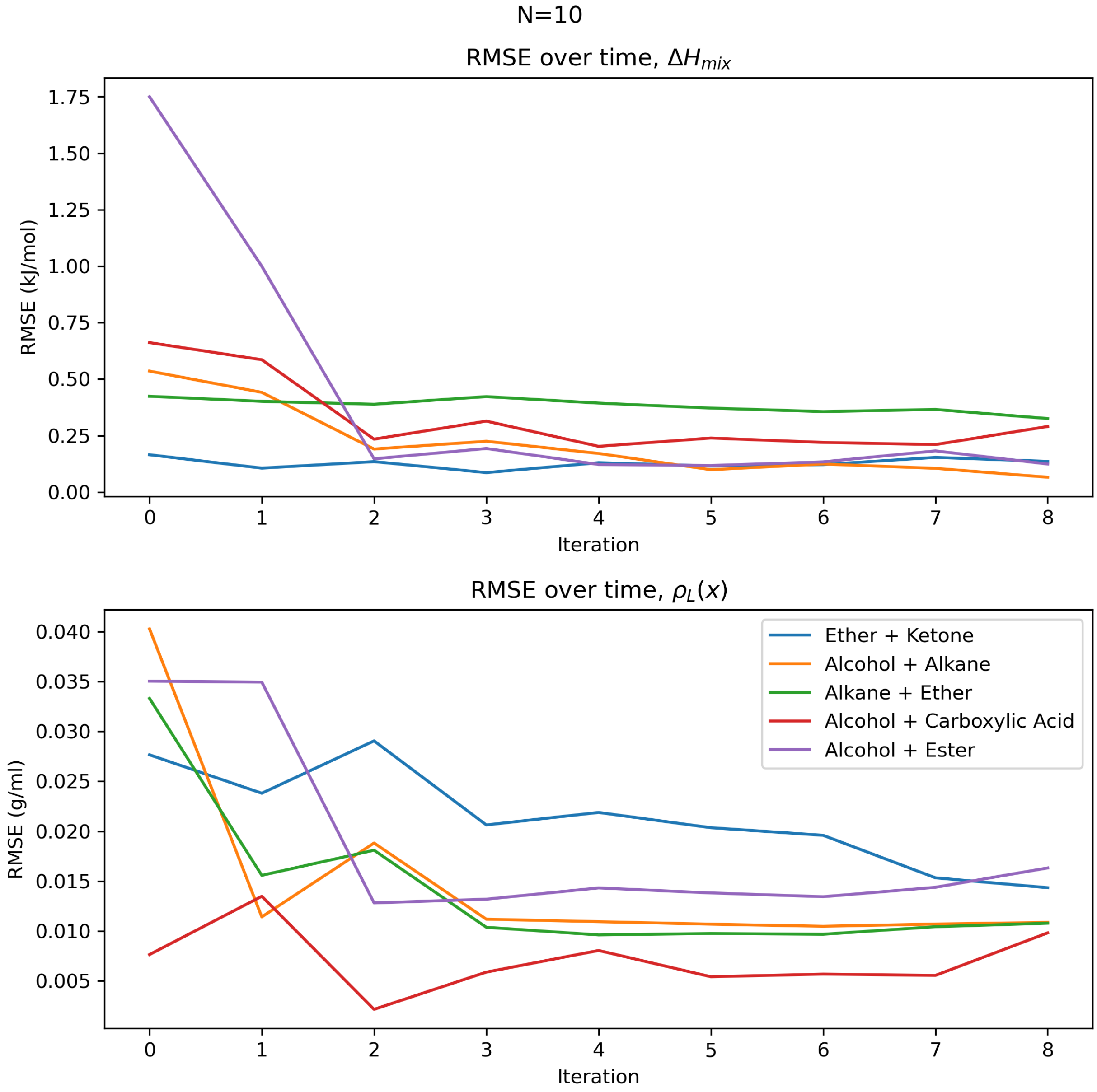

Figure 30. Per-moiety training set RMSE at each accepted optimization step, for N=10 multi-fidelity optimization against "mixture-only" training set.

2.4 “Mixture Only” optimization, N=20

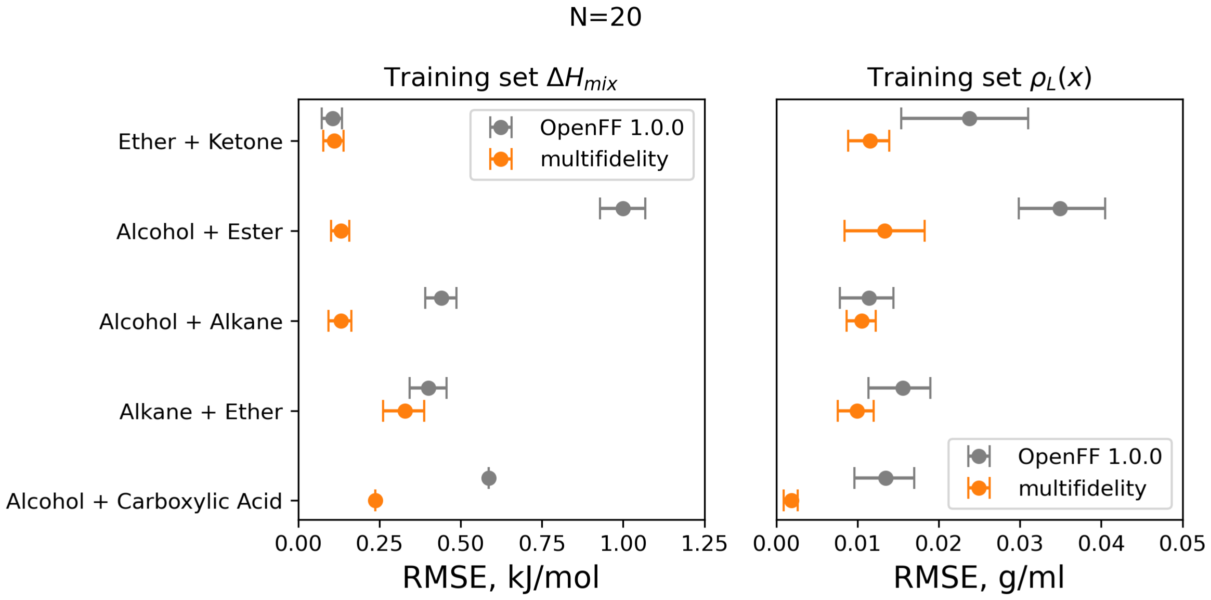

Figure 31. RMSE in training set ΔH_{mix} and $\rho_L(x)$, for OpenFF 1.0.0 and retrained parameters from N=20 initial points multi-fidelity optimization against “mixture-only” training set. Error bars represent bootstrapped 95% confidence intervals

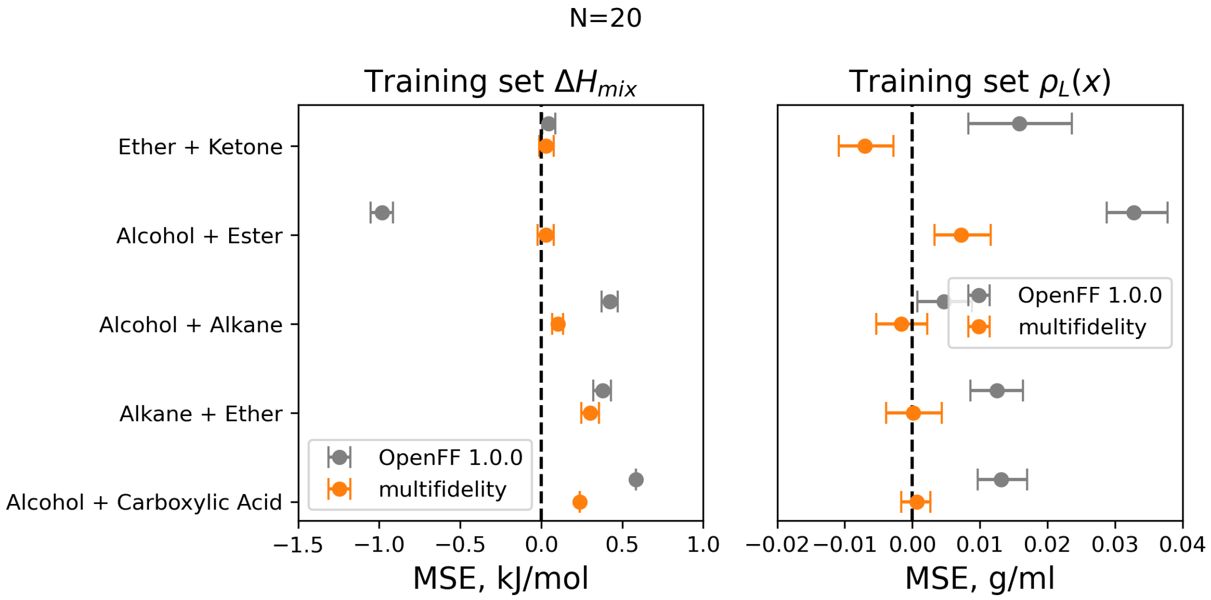

Figure 32. Bias in training set ΔH_{mix} and $\rho_L(x)$, as measured by the mean signed deviation (MSD), for OpenFF 1.0.0 and retrained parameters from N=20 multi-fidelity optimization against “mixture-only” training set. Error bars represent bootstrapped 95% confidence intervals

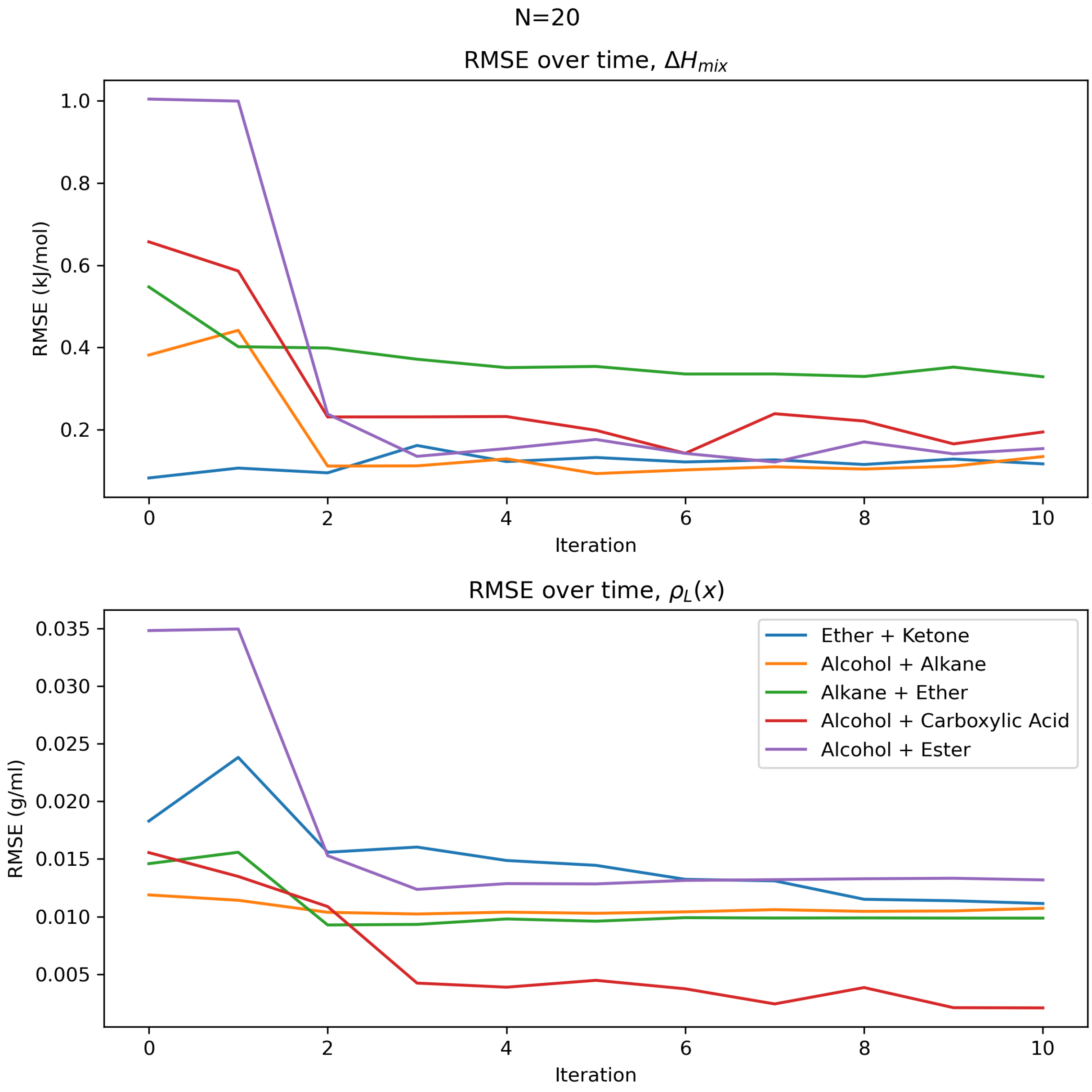

Figure 33. Per-moiety training set RMSE at each accepted optimization step, for N=20 multi-fidelity optimization against "mixture-only" training set.

3 Test set performance

3.1 “Pure only”, N=10

3.1.1 Run 1

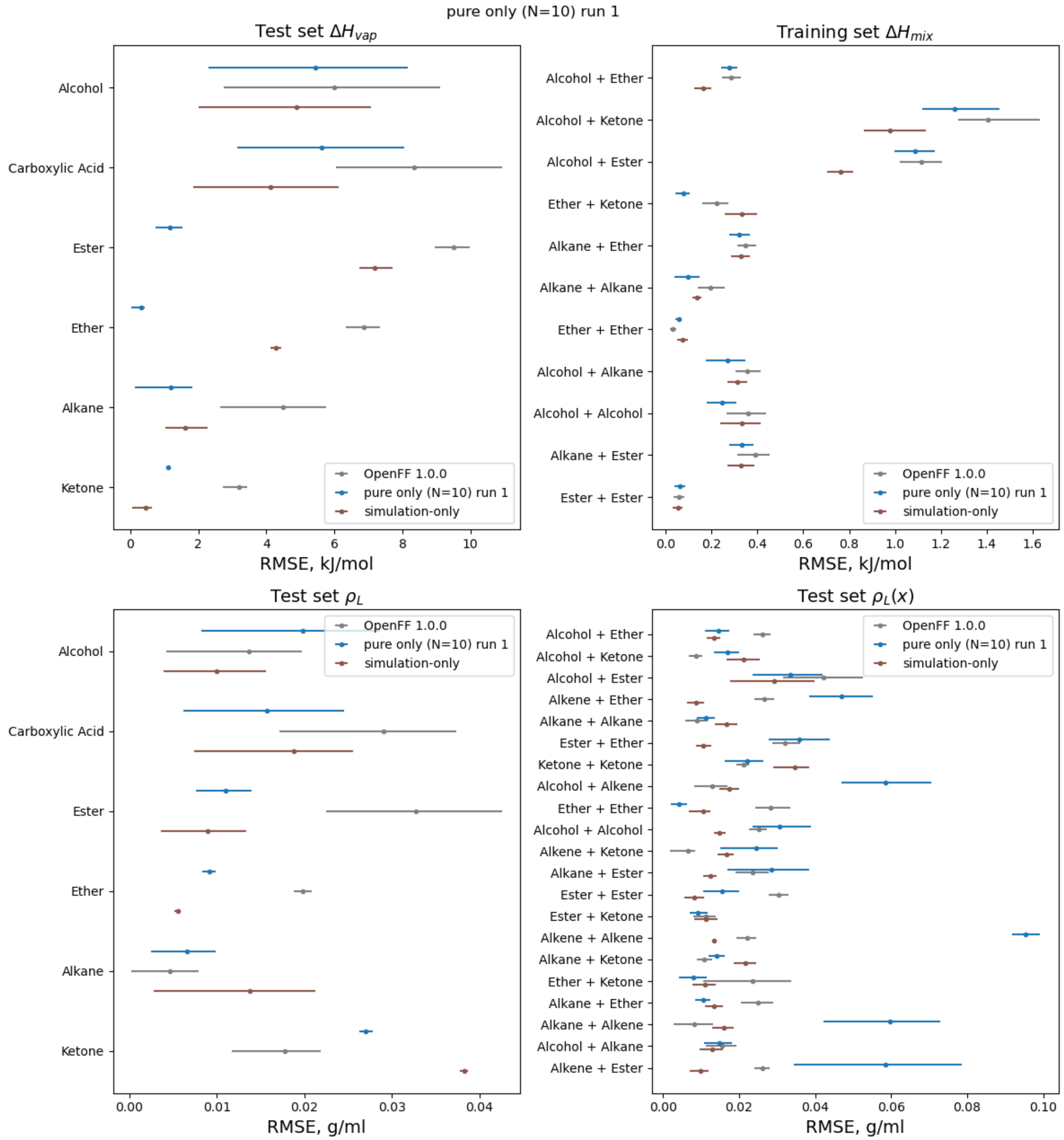

Figure 34. Benchmark RMSE over the four types of physical property data in the test set, split by function group or functional group mixture. RMSEs plotted for OpenFF 1.0.0 (gray), multi-fidelity N=10 run 1 against the “pure only” training set (blue), and the simulation-only optimization against the same training set (brown). Error bars represent 95% confidence intervals, bootstrapped over the properties in the dataset.

3.1.2 Run 2

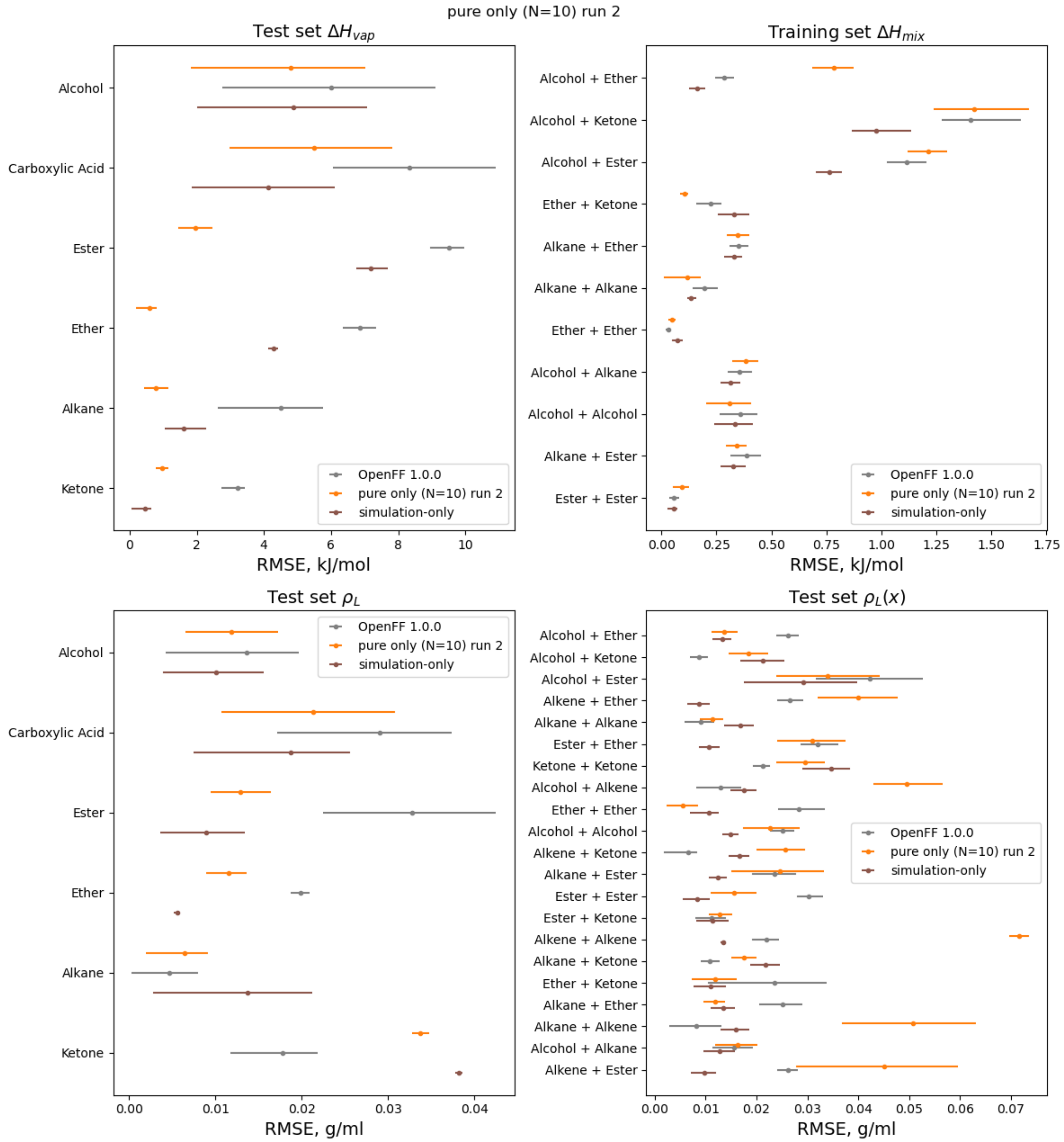

Figure 35. Benchmark RMSE over the four types of physical property data in the test set, split by function group or functional group mixture. RMSEs plotted for OpenFF 1.0.0 (gray), multi-fidelity N=10 run 2 against the "pure only" training set (orange), and the simulation-only optimization against the same training set (brown). Error bars represent 95% confidence intervals, bootstrapped over the properties in the dataset.

3.1.3 Run 3

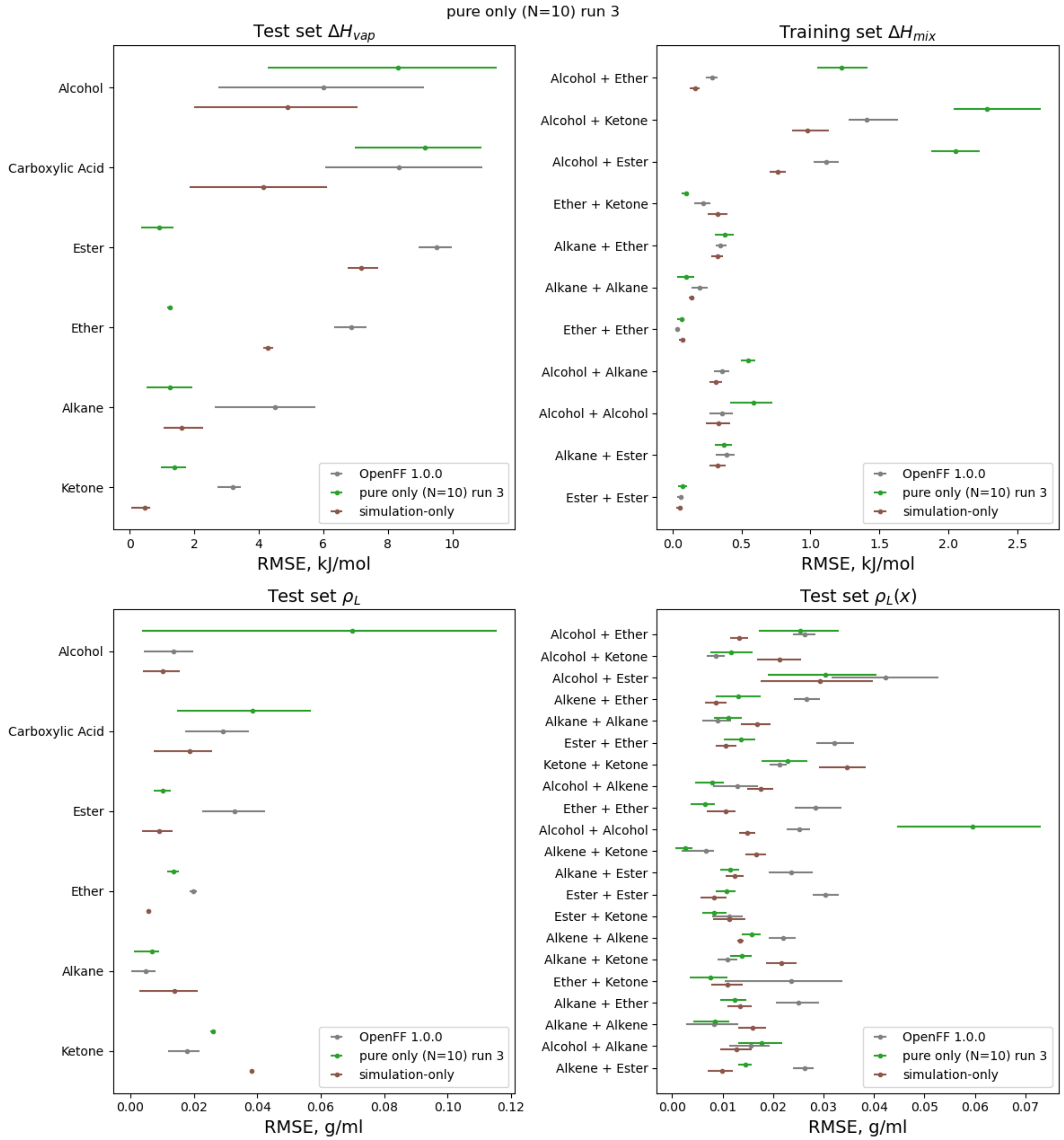

Figure 36. Benchmark RMSE over the four types of physical property data in the test set, split by function group or functional group mixture. RMSEs plotted for OpenFF 1.0.0 (gray), multi-fidelity N=10 run 3 against the “pure only” training set (green), and the simulation-only optimization against the same training set (brown). Error bars represent 95% confidence intervals, bootstrapped over the properties in the dataset.

3.1.4 Run 4

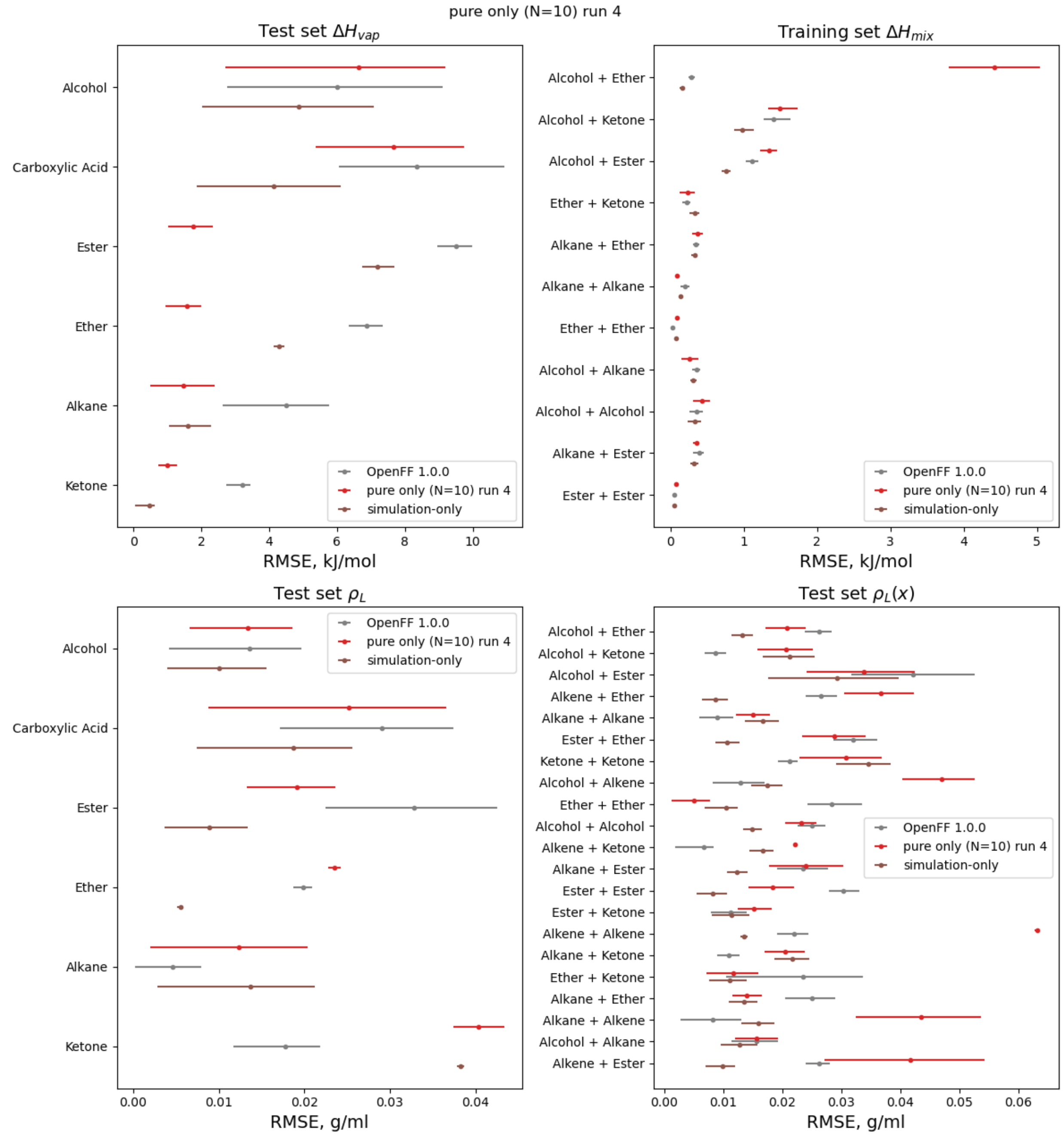

Figure 37. Benchmark RMSE over the four types of physical property data in the test set, split by function group or functional group mixture. RMSEs plotted for OpenFF 1.0.0 (gray), multi-fidelity N=10 run 4 against the "pure only" training set (red), and the simulation-only optimization against the same training set (brown). Error bars represent 95% confidence intervals, bootstrapped over the properties in the dataset.

3.1.5 Run 5

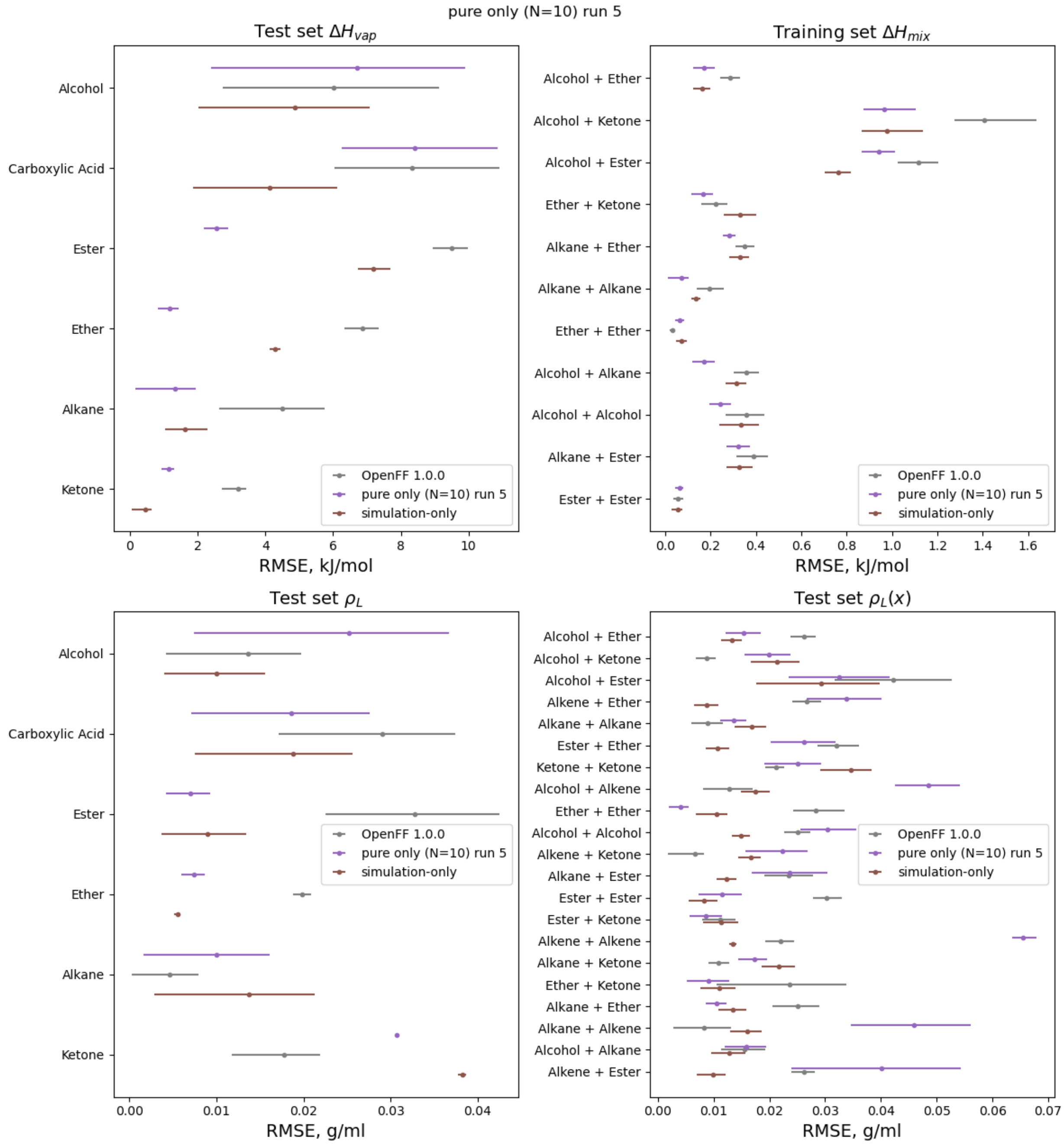

Figure 38. Benchmark RMSE over the four types of physical property data in the test set, split by function group or functional group mixture. RMSEs plotted for OpenFF 1.0.0 (gray), multi-fidelity N=10 run 5 against the "pure only" training set (purple), and the simulation-only optimization against the same training set (brown). Error bars represent 95% confidence intervals, bootstrapped over the properties in the dataset.

3.2 “Mixture only”, N=10

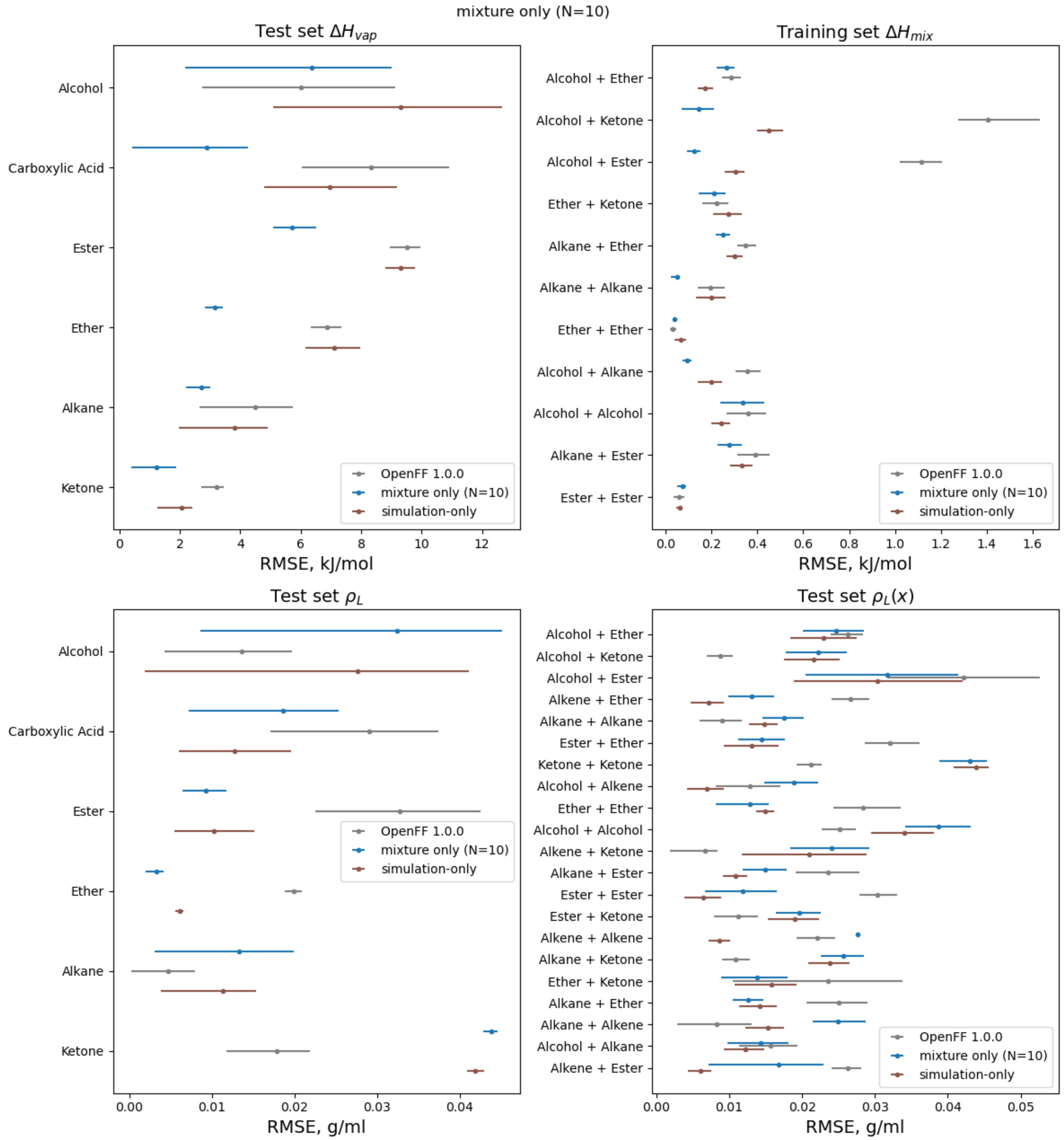

Figure 39. Benchmark RMSE over the four types of physical property data in the test set, split by function group or functional group mixture. RMSEs plotted for OpenFF 1.0.0 (gray), multi-fidelity N=10 run against the “mixture only” training set (blue), and the simulation-only optimization against the same training set (brown). Error bars represent 95% confidence intervals, bootstrapped over the properties in the dataset.

3.3 “Mixture only”, N=20

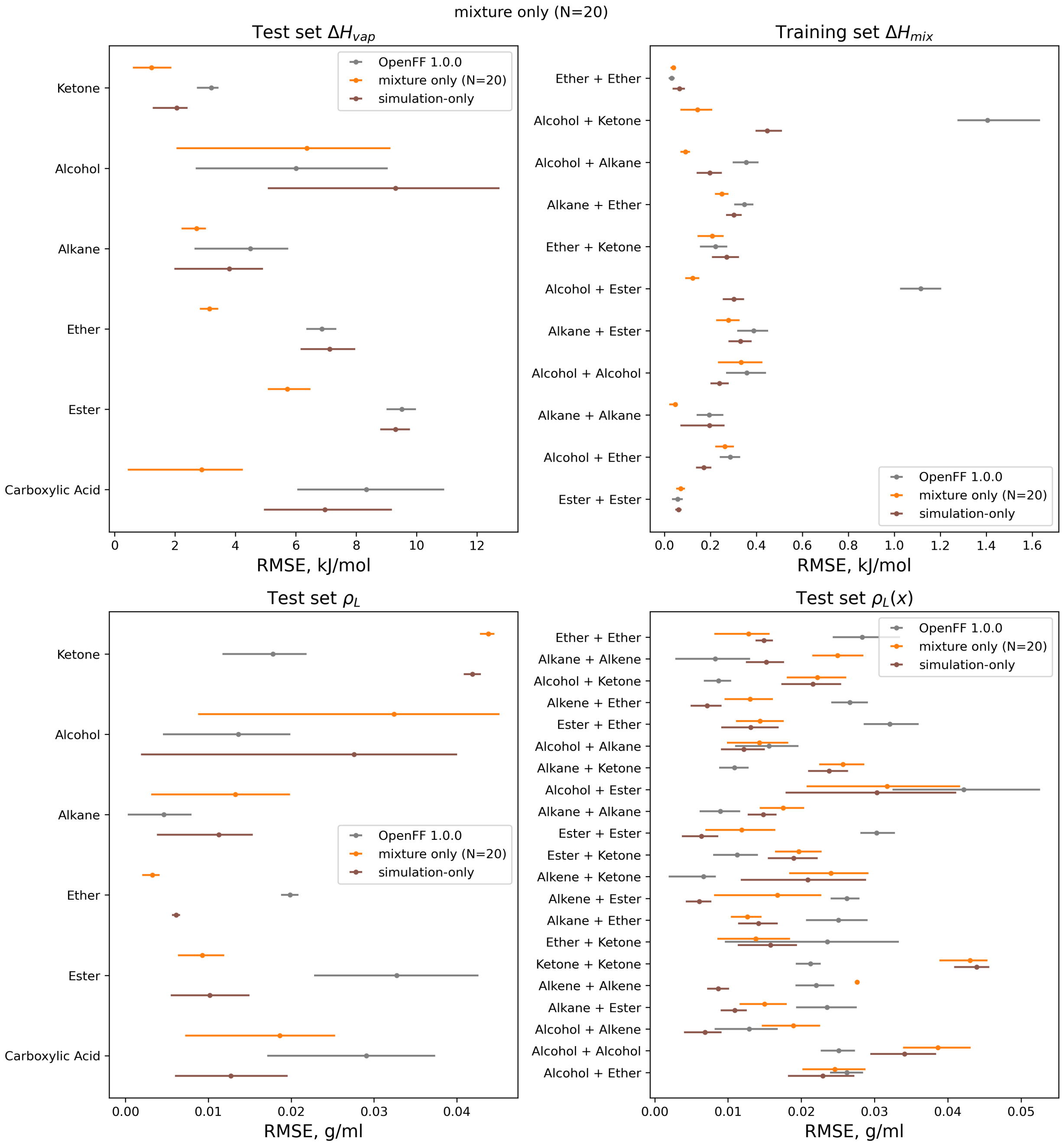

Figure 40. Benchmark RMSE over the four types of physical property data in the test set, split by function group or functional group mixture. RMSEs plotted for OpenFF 1.0.0 (gray), multi-fidelity N=20 run against the “mixture only” training set (blue), and the simulation-only optimization against the same training set (brown). Error bars represent 95% confidence intervals, bootstrapped over the properties in the dataset.
